# Novel Furfural-Derived Polyaldimines as Latent Hardeners for Polyurethane Adhesives

**DOI:** 10.1021/acsami.3c17416

**Published:** 2024-01-29

**Authors:** Tankut Türel, Berend Eling, Anna M. Cristadoro, Thomas Mathieu, Martin Linnenbrink, Željko Tomović

**Affiliations:** † Polymer Performance Materials Group, Department of Chemical Engineering and Chemistry, Eindhoven University of Technology, 5600 MB Eindhoven, The Netherlands; ‡ Institute of Technical and Macromolecular Chemistry, University of Hamburg, Bundesstrasse 45, 20146 Hamburg, Germany; § BASF Polyurethanes, Elastogranstrasse 60, 49448 Lemfoerde, Germany; ∥ Institute for Complex Molecular Systems, Eindhoven University of Technology, 5600 MB Eindhoven, The Netherlands

**Keywords:** latent hardener, polyurethane, imine, one-component system, adhesive

## Abstract

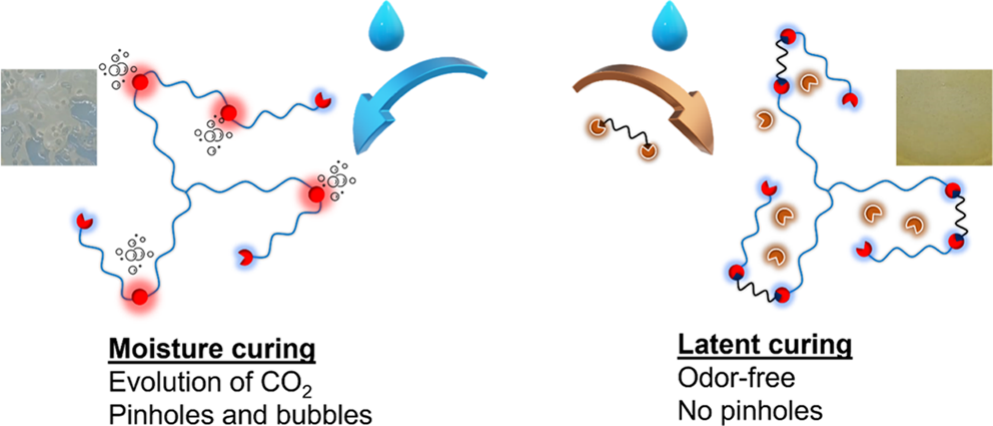

Moisture-curing one-component polyurethane systems in adhesive, sealant, and coating applications may show blister formation upon cure. Blisters can be formed when carbon dioxide, generated in the reaction with isocyanate and water, is trapped in the film. This problem can be mitigated by employing latent hardeners such as blocked polyamines, which are activated upon moisture exposure. The hydrolysis of the latent hardener yields the polyamine that quickly reacts with the isocyanate, forming urea linkages, and then chain extends the polymer. The hydrolysis also releases the blocking agent, which can potentially create an unpleasant odor. In this work, a series of di- and trifunctional aldimines were synthesized from commercially available polyamines, biobased hydroxymethyl furfural, and lauroyl chloride. Hydroxymethyl furfural was first esterified with lauroyl chloride and subsequently condensed with the polyamines to form the aldimines. The application of these novel aldimines in a model moisture-curing system allowed the preparation of blister- and odor-free castings. Based on our results, the mechanical performance of the different aldimines in casting and adhesive applications could be related to the polymer network density. This was dependent on the rate of the aldimine hydrolysis reaction to produce the polyamine. In particular, the use of aldimines prepared from polyether amines and 1,5-diamino-2-methylpentane showed excellent adhesive properties.

## Introduction

Polyurethanes (PU) represent a versatile class of polymers, widely utilized across various industries due to their remarkable properties and diverse applications.^1−3^ Flexible and rigid foams^4,5^ are the dominant sectors, followed by high-performance adhesives,^6,7^ coatings,^8,9^ sealants,^10,11^ and elastomers.^12^ Furthermore, they are pivotal components in key industry sectors such as the automotive,^13^ construction,^14^ and textiles.^15^

PU systems for adhesives can be broadly categorized into two groups: 1K (one-component) and 2K (two-component) systems. While these categories share certain similarities, each offers its own distinct set of advantages and disadvantages.^16^ Traditionally, 2K PU systems have been recognized as the superior technology because of high formulation freedom and improved mechanical properties. Nevertheless, the 2K systems require storage and handling of the two different reaction components. Furthermore, it is crucial to thoroughly mix the two components and specialized equipment may be needed.^1,17^ Its major drawback, however, is the limited working time once mixed.^3^ To address these challenges, 1K PU systems have emerged as an alternative, offering a solution that eliminates the aforementioned handling issues while delivering adequate performance.^17^ These systems are prepolymers based on reacting a polyether or polyester polyol with a molar excess of di-isocyanate.^1^ The curing reaction is between isocyanate and water to form urea and carbon dioxide. Because the required amount of water comes from the environment, these adhesives are also termed moisture-cured adhesives. For successful curing, it is important that moisture can diffuse into the bond, and if atmospheric moisture is needed, then the joint must be designed to allow water diffusion, or at least one of the substrates must be porous. Moisture-cured adhesives are used in construction applications to bond porous materials such as wood, which can contain some humidity intrinsically, with each other (i.e., wood–wood) or with nonporous substrates such as metal or plastic sheeting material (e.g., metal–wood). The carbon dioxide formation, however, may give rise to blister formation and poor adhesion.^18,19^

**Figure 1:**
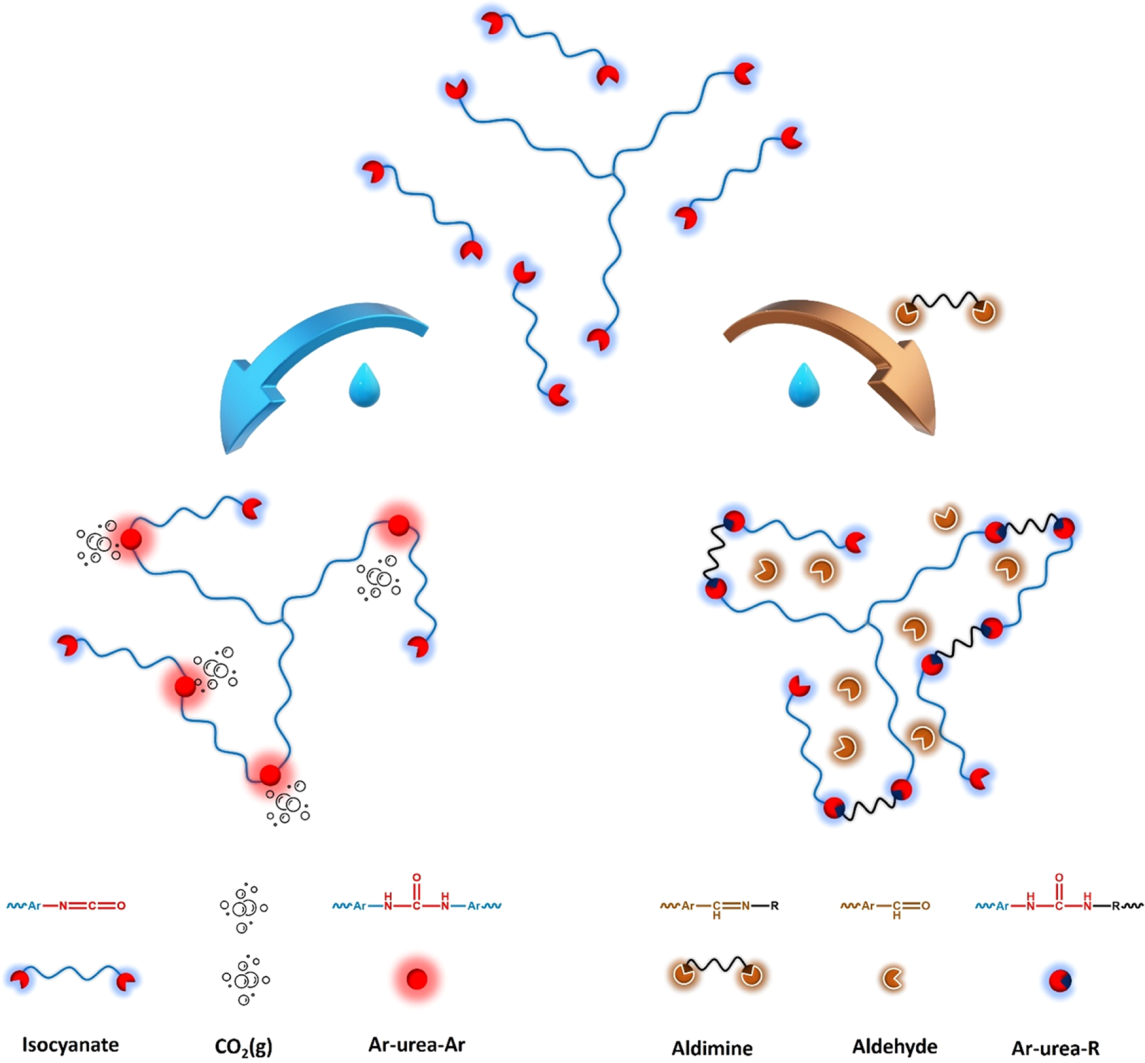
Illustration of the curing principle of isocyanate prepolymer with and without adding an aldimine as a latent curing agent in a humid environment, where Ar–urea–R stands for the urea linkage, which is formed through the reaction of released amine and free isocyanate, and Ar–urea–Ar represents urea formed due to the reaction of isocyanate with water. More information can be found in the Supporting Information (Scheme S1).

**Scheme 1:**
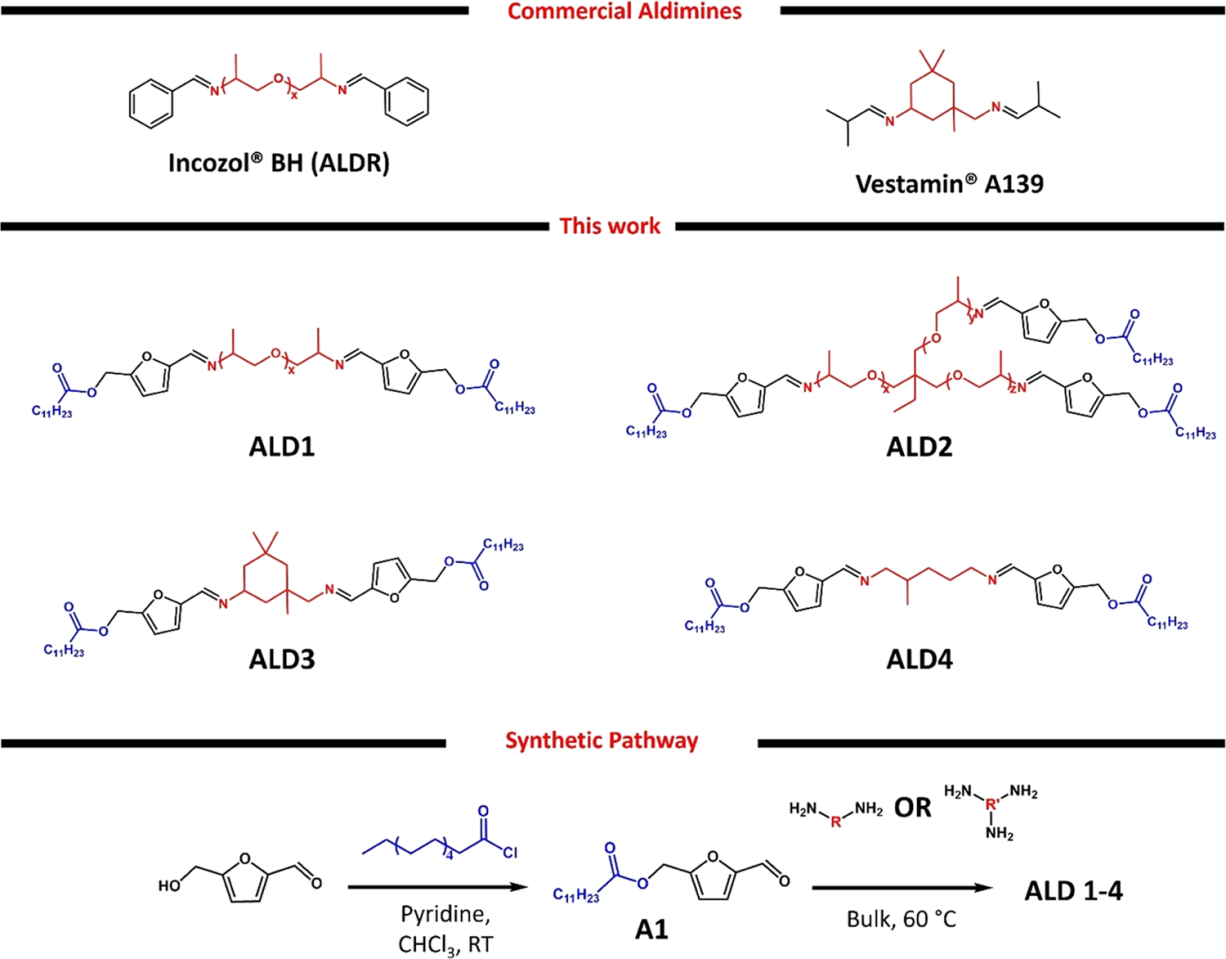
Structures of the Commercially Available Aldimines and the Aldimines Developed in This Work and the Synthetic Pathway toward the Novel Aldimines **ALD1–4** R denotes Jeffamine D230, isophorone diamine, and 1,5-diamino-2-methylpentane for **ALD1**, **ALD3**, and **ALD4**, respectively; R′ is Jeffamine T 403 for **ALD2**.

To mitigate the adverse effects of gas formation, latent hardeners have been developed that become active when exposed to moisture.^20−26^ Typical examples of latent hardeners for curing of 1K PU systems are blocked amines, such as imines and oxazolidines. The commercially available latent hardeners Incozol BH and Vestamin A139 are aldimine-based (Scheme 1) and widely used in the PU coating, sealants, and adhesive industry.^27,28^ These latent hardeners that contain more than one aldimine group per molecule hydrolyze readily, and the polyamine generated reacts fast with the isocyanate prepolymer. The combined hydrolysis and chain extension reaction exceeds the reaction of water with the isocyanate (Figure 1 and Scheme S1), and hence, the rate of gas formation is suppressed and its amount reduced. The hydrolysis also generates an aldehyde such as benzaldehyde or isobutyraldehyde in the case of Incozol BH and Vestamin A139, respectively.

The release of small-molecular-weight aldehydes may cause smell problems. In order to overcome such problems, the incorporation of aldimines comprising high-molecular-weight aldehydes has been pursued.^29,30^ For instance, Burckhardt and collaborators developed a range of aliphatic aldimines based on 2,2-dimethyl-3-lauroyloxypropanal.^29^ However, the preparation of such an aldehyde is not straightforward due to the tendency of 2,2-dimethyl-3-hydroxypropanal to undergo dimerization during the synthesis process.^31,32^ In another investigation by the same research group, they explored a series of aromatic aldimines.^30^ Nevertheless, the synthesis of the constituent aldehydes poses considerable difficulty, requiring the use of a toxic catalyst and resulting in a mixture of products. Additives containing multiple oxazolidine rings are also applied as latent hardeners. The hydrolysis releases acetone instead of carbon dioxide, which potentially also may lead to blistering issues.^33^

Aldimines represent promising candidates as latent hardeners because of their straightforward synthesis without the need for purification, chemical inertness toward isocyanates, and rapid response to the humid environment, thereby releasing the amine hardener. We have developed several aldimine-based latent hardeners by utilizing the ester of biobased 5-hydroxymethyl furfural (HMF) and lauroyl chloride as a blocking agent (Scheme 1). Apart from its biobased origin, HMF is both cost-effective and readily available. It possesses two functional groups, aldehyde and hydroxyl, that facilitate direct and straightforward functionalization. Additionally, its aromatic nature prevents imine tautomerization due to the absence of α-hydrogen. The performance of the aldimines was evaluated in a model 1K polyether-MDI prepolymer system. The elastomer and adhesive properties of these aldimine-modified systems were studied. The castings prepared in the presence of the novel aldimines were blister- and odor-free. The mechanical properties could be related to the cross-link density of the formed polymer, which in turn was determined by the amine-functionality and the hydrolysis rate of the aldimines.

## Experimental Section

### Materials

5-Hydroxymethylfurfural (HMF, 98%) was procured from Manchester Organics and used without further purification. Lauroyl chloride (98%), *p*-tolylisocyanate (99%), 1,4-diazabicyclo [2.2.2]octane (DABCO, ≥99%), Jeffamine D230 (*M*_n_ ∼ 230), Jeffamine T403 (*M*_n_ ∼ 440), isophorone diamine (IPDA, ≥99%), 1,5-diamino-2-methylpentane (99%), and benzoic acid (≥99.5%) were purchased from Merck. Deuterated CDCl_3_ was obtained from Cambridge Isotope Laboratories; THF-*d*_8_ was from Merck. Ethyl acetate, chloroform, dichloromethane, tetrahydrofuran, and pyridine were procured from Biosolve B.V. and used without further purification. Methylene diphenyl diisocyanate (4,4′-MDI, Lupranat ME), Lupranol 1005/1 (polypropylene glycol with an average molecular weight of 4000 and OH number of 28 mg KOH/g), and Lupranol 2095 (trifunctional reactive polyether polyol containing primary hydroxyl groups with OH number of 35 mg KOH/g) were obtained from BASF (Germany) and used as received. The amine functionalities of Jeffamine D230 and Jeffamine T403 were determined to be 1.7 and 2.2, respectively, using titrimetric ^1^H NMR analysis. The amine values of Jeffamine D230 and T403 amounted to 414.7 and 280.5 mg of KOH/g, respectively.

### Methods

The ^1^H and ^13^C NMR spectra were recorded on a Bruker UltraShield (400 MHz) using CDCl_3_ or THF-*d*_8_ as the solvent. Mass spectroscopy of the compounds was performed with a Bruker AutoflexIII TOF/TOF MALDI analyzer. The Fourier-transform infrared (FTIR) spectra were recorded on a Thermo Scientific NICOLET iS20 FTIR spectrometer as an average of 8 scans over the wavenumber range of 4000–450 cm^–1^.

To monitor the hydrolysis process using ^1^H NMR spectroscopy, stock solutions of the various components in THF-*d*_8_ were prepared. The concentrations of the water and *p*-tolyl isocyanate solution were set at 0.2 M in THF-*d*_8_, while the benzoic acid mixture was composed of 0.2 M water and 0.1 M benzoic acid in THF-*d*_8_. The aldimine concentration was maintained at 0.1 M in THF-*d*_8_. For the uncatalyzed systems, equal volumes of the water solution, aldimine solution, and *p*-tolyl isocyanate solution were combined. Equal volumes of the benzoic acid/water mixture, aldimine solution, and *p*-tolyl isocyanate solution were mixed for the catalyzed systems. During the experiment, the data collection process was initiated by performing ^1^H NMR measurements every 5 min for the first 30 min. Subsequently, hourly measurements were conducted for a total duration of 24 h. Similarly and at identical concentrations, we performed a control experiment using *p*-tolylisocyanate and water without the addition of aldimines.

The isocyanate content of the prepolymer was determined using a Metrohm 916 Ti-Touch titrator according to the ASTM D5155-19 standard method.

Dynamic mechanical analysis (DMA) measurements were performed on a TA Instruments DMA850. Storage and loss moduli (*E*′ and *E*″) were measured as a function of temperature. The experiments were carried out from −80 to 150 °C at a heating rate of 3 °C/min under an oscillatory strain of 0.1% and a frequency of 1 Hz with a preload force of 0.05 N. Glass-transition temperatures (*T*_g_) were determined from the maxima of the loss modulus curves.

The tensile tests were performed with a Zwick/Roell Intelligent testing machine at a strain rate of 10 mm/min and a preload of 0.05 N. The true stress of materials was determined using the software TestXpert III-V1.61. The Young modulus of the materials was determined by calculating the slope of the derivative of the stress–strain curves from 1 to 10% strain. The experimental error in tensile and elongation at break was about 10%.

Lap-shear tests were performed according to ISO 4587/DIN EN 1465 at a strain rate of 5 mm/min, utilizing a preforce of 10 N. The experimental error in the lap-shear strength was about 15%.

Swelling experiments were performed with tetrahydrofuran (THF). The swelling ratio was calculated using eq 1, where *q* represents the swelling ratio, *W*_0_ is the initial weight of polymer, and *W*_s_ is the weight of swollen network.^34^
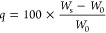
1Gel fractions were calculated using eq 2, where ϕ stands for gel fraction, *W*_0_ is the initial weight of the polymer, and *W*_1_ is the weight after drying.^34^
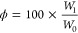
2Cross-linking densities of the networks (*ν*_e_) were calculated using eq 3:^35^
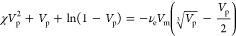
3where *ν*_p_ is the polymer volume fraction, *V*_m_ is the molar volume of the solvent (THF = 81.0 mL/mol), χ is the polymer–solvent interaction parameter.^35^ The latter can be calculated using eq 4:^36^
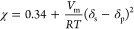
4where δ_s_ and δ_p_ are the solubility parameters of THF (δ_s_ = 18.0 MPa^0.5^) and PU polymer, respectively. The solubility parameter of the polymer was taken as that reported for polypropylene glycol (δ_p_ = 17.5 ± 2.5 MPa^0.5^).^37^ χ was taken as 0.34.

### Synthesis

#### Aldehyde **A1**

The synthesis method was adopted from the literature.^38^ 5-Hydroxymethylfurfural (HMF, 50.0 g, 396.5 mmol) was dissolved in chloroform (300 mL) and pyridine (40 mL) under Ar in a three-neck round-bottom flask. After purging with Ar for 30 min, lauroyl chloride (86.7 g, 396.5 mmol) was added, and the mixture was allowed to mix for 24 h at room temperature. After completion of the reaction, the product was extracted twice with 1 M HCl (100 mL) and once with deionized water (100 mL). The organic layer was dried over magnesium sulfate (MgSO_4_). Finally, aldehyde **A1** was obtained as a white powder with a melting point of 55 °C after evaporation of chloroform using a rotary evaporator. ^1^H NMR (400 MHz, CDCl_3_) δ: 9.64 (s, 1H), 7.21 (d, *J* = 3.6 Hz, 1H), 6.58 (d, *J* = 3.6 Hz, 1H), 5.13 (s, 2H), 2.35 (t, *J* = 7.5 Hz, 2H), 1.63 (p, *J* = 7.1 Hz, 2H), 1.26 (m, 16H), 0.88 (t, *J* = 6.8 Hz, 3H). ^13^C NMR (101 MHz, CDCl_3_) δ: 177.93, 173.30, 155.82, 152.94, 121.76, 112.58, 57.76, 34.10, 32.02, 29.71, 29.69, 29.55, 29.44, 29.33, 29.18, 24.93, 22.80, 14.23. MS (MALDI-TOF) *m*/*z*: [M + Na^+^]^+^ calculated for C_18_H_28_NaO_4_^+^ 331.19, found 331.18.

#### Aldimine **ALD1**

Aldehyde **A1** (2.0 g, 6.5 mmol) was mixed with Jeffamine D230 (0.88 g). The reaction was carried out at 60 °C at 15 mbar for 2 h using a rotary evaporator and continued for 12 h under vacuum at 40 °C. **ALD1** was obtained as a light-brown liquid. ^1^H NMR (400 MHz, CDCl_3_) δ: 8.14–7.99 (m, 2H), 6.69 (m, 2H), 6.46 (m, 2H), 5.08 (s, 4H), 3.99–2.87 (m, 13H), 2.32 (t, *J* = 7.6 Hz, 4H), 1.61 (p, *J* = 7.3 Hz, 4H), 1.34–0.99 (m, 45H), 0.88 (t, *J* = 6.7 Hz, 6H). ^13^C NMR (101 MHz, CDCl_3_) δ: 173.48, 152.10, 149.42, 115.36, 112.32, 75.35, 73.58, 66.49, 58.03, 34.20, 32.02, 29.71, 29.55, 29.44, 29.35, 29.22, 24.94, 22.80, 18.87, 17.40, 14.23.

#### Aldimine **ALD2**

Aldehyde **A1** (2.0 g, 6.5 mmol) was reacted with Jeffamine T403 (1.30 g) as before. **ALD2** was obtained as a light brown liquid. ^1^H NMR (400 MHz, CDCl_3_) δ: 8.15–7.92 (m, 3H), 6.69 (m, 3H), 6.46 (m, 3H), 5.08 (s, 6H), 3.97–2.82 (m, 31H), 2.31 (m, 6H), 1.61 (p, *J* = 7.4, 7.0 Hz, 6H), 1.47–0.71 (m, 79H). ^13^C NMR (101 MHz, CDCl_3_) δ: 173.50, 152.13, 149.40, 115.26, 112.31, 75.62, 73.59, 71.77, 66.67, 58.06, 43.95, 34.22, 32.04, 29.72, 29.57, 29.46, 29.37, 29.24, 24.96, 22.81, 18.99, 17.57, 14.25, 7.58.

#### Aldimine **ALD3**

Aldehyde **A1** (2.0 g, 6.5 mmol) was reacted with the isophorone diamine (0.55 g, 3.24 mmol) as before. **ALD3** was obtained as a brown liquid. ^1^H NMR (400 MHz, CDCl_3_) δ: 8.21–7.84 (m, 2H), 6.76–6.60 (m, 2H), 6.46 (m, 2H), 5.08 (m, 4H), 3.79–3.19 (m, 3H), 2.32 (m, 4H), 1.73 (s, 3H), 1.61 (m, 4H), 1.48 (m, 3H), 1.26 (m, 32H), 1.19 (s, 2H), 1.10 (s, 3H), 0.97 (m, 4H), 0.88 (t, *J* = 6.8 Hz, 6H). ^13^C NMR (101 MHz, CDCl_3_) δ: 173.55, 173.53, 152.32, 152.17, 152.16, 152.13, 152.08, 151.87, 151.79, 150.31, 150.06, 148.12, 148.07, 114.93, 114.41, 113.86, 112.33, 69.95, 63.96, 63.75, 58.05, 48.25, 48.14, 47.18, 47.11, 43.42, 43.35, 36.69, 36.67, 35.46, 35.43, 34.24, 32.04, 31.75, 31.63, 30.79, 29.72, 29.57, 29.46, 29.36, 29.23, 28.31, 25.01, 24.97, 22.82, 14.25. MS (MALDI-TOF) *m*/*z*: [M + H^+^]^+^ calculated for C_46_H_75_N_2_O_6_^+^ 751.56, found 751.55.

#### Aldimine **ALD4**

Aldehyde **A1** (4.89 g, 15.9 mmol) was reacted with 1,5-diamino-2-methylpentane (0.92 g, 7.9 mmol) as before. **ALD4** was obtained as a yellowish powder with a melting temperature of 45 °C. ^1^H NMR (400 MHz, CDCl_3_) δ 8.03 (d, *J* = 13.6 Hz, 2H), 6.68 (t, *J* = 3.7 Hz, 2H), 6.47 (d, *J* = 3.4 Hz, 2H), 5.08 (s, 4H), 3.57 (tq, *J* = 11.6, 7.0, 6.1 Hz, 3H), 3.32 (dd, *J* = 11.5, 7.3 Hz, 1H), 2.32 (t, *J* = 7.5 Hz, 4H), 1.97 (dt, *J* = 13.5, 7.5 Hz, 1H), 1.78 (m, 2H), 1.61 (p, *J* = 7.0 Hz, 4H), 1.53–1.39 (m, 1H), 1.26 (m, 34H), 0.93 (d, *J* = 6.6 Hz, 3H), 0.88 (t, *J* = 6.7 Hz, 6H). ^13^C NMR (101 MHz, CDCl_3_) δ: 173.41, 151.98, 151.97, 151.95, 151.92, 149.79, 149.52, 114.65, 114.52, 112.24, 68.66, 62.25, 57.94, 34.14, 32.53, 31.96, 29.65, 29.64, 29.49, 29.39, 29.29, 29.15, 28.30, 24.89, 22.74, 18.11, 14.18. MS (MALDI-TOF) *m*/*z*: [M + H^+^]^+^ calculated for C_42_H_69_N_2_O_6_^+^ 697.52, found 697.51.

#### Aldimine **ALDR** (Commercially Available as Incozol BH)

Benzaldehyde (2.0 g, 18.8 mmol) was reacted with Jeffamine D230 (2.55 g) as before. **ALDR** was obtained as a yellow-colored liquid. ^1^H NMR (400 MHz, CDCl_3_) δ: 8.36–8.19 (m, 1H), 7.79–7.67 (m, 2H), 7.39 (dq, *J* = 5.3, 2.3 Hz, 3H), 3.73–2.98 (m, 7H), 1.34–0.90 (m, 5H). ^13^C NMR (100 MHz, CDCl_3_) δ: 160.57, 136.53, 130.52, 128.96, 128.24, 76.01, 75.31, 74.36, 73.89, 67.15, 18.95, 17.44.

#### Prepolymer Synthesis

The reference prepolymer (**PPR1**) was prepared from Lupranol 2095 (25.85 g) and Lupranol 1005/1 (12.95 g). The two polyols were mixed with 56 mg of DABCO and heated to 80 °C under an Ar flow. Subsequently, a mixture of aldehyde **A1** (4.13 g) and 4,4′-MDI (6.2 g) was added at once and stirred for 3 h. The experimental NCO content (2.34%) was close to the theoretical value (2.30%). The prepolymers with aldimines were prepared similarly with the exception that **A1** was replaced by 6.6 mmol of **ALD1**, **ALD3**, **ALD4**, and **ALDR** and 4.4 mmol of **ALD2** (Table S1), respectively.

### Sample Preparation

For the catalyzed systems, 4 g of isocyanate prepolymer containing approximately 1 mmol of imine groups was poured into an oven-dried poly(tetrafluoroethylene) (PTFE) mold with a diameter of 6 cm. Subsequently, 0.2 mmol of benzoic acid (0.2 equiv relative to the imine groups) in THF (1 mL) was added and well-stirred until homogeneity and evacuated for 3 min to remove the bubbles and THF. The casts were cured for 1 week at 23 °C and 60% relative humidity (RH) and had a thickness of about 1.5 mm. The reactivity of the system was expressed as tack-free time, the time required for the sample to obtain a dry surface. The experimental error in the time-free determination of tack is about 15%. The uncatalyzed systems were prepared in the same manner, albeit without benzoic acid. Photographs of the produced films are shown in Table S2. Bubble formation was assessed semiquantitatively based on the number of bubbles that were formed during curing. The odor was assessed qualitatively by smelling at a distance of 10 cm after 7 days of curing at 23 °C and 60% relative humidity. Subsequently, dumbbell-shaped specimens (effective length, width, and thickness: 12, 2, and 1.5 mm, respectively) were prepared for tensile testing.

The adhesion tests were performed using beechwood test bars (2.5 cm × 10 cm, obtained from Rocholtt, Germany). The specimen overlap was 25 mm × 25 mm, and the thickness of the adhesive layer was kept at about 100 μm using a spacer.^39^

## Results and Discussion

### Synthesis

**Figure 2:**
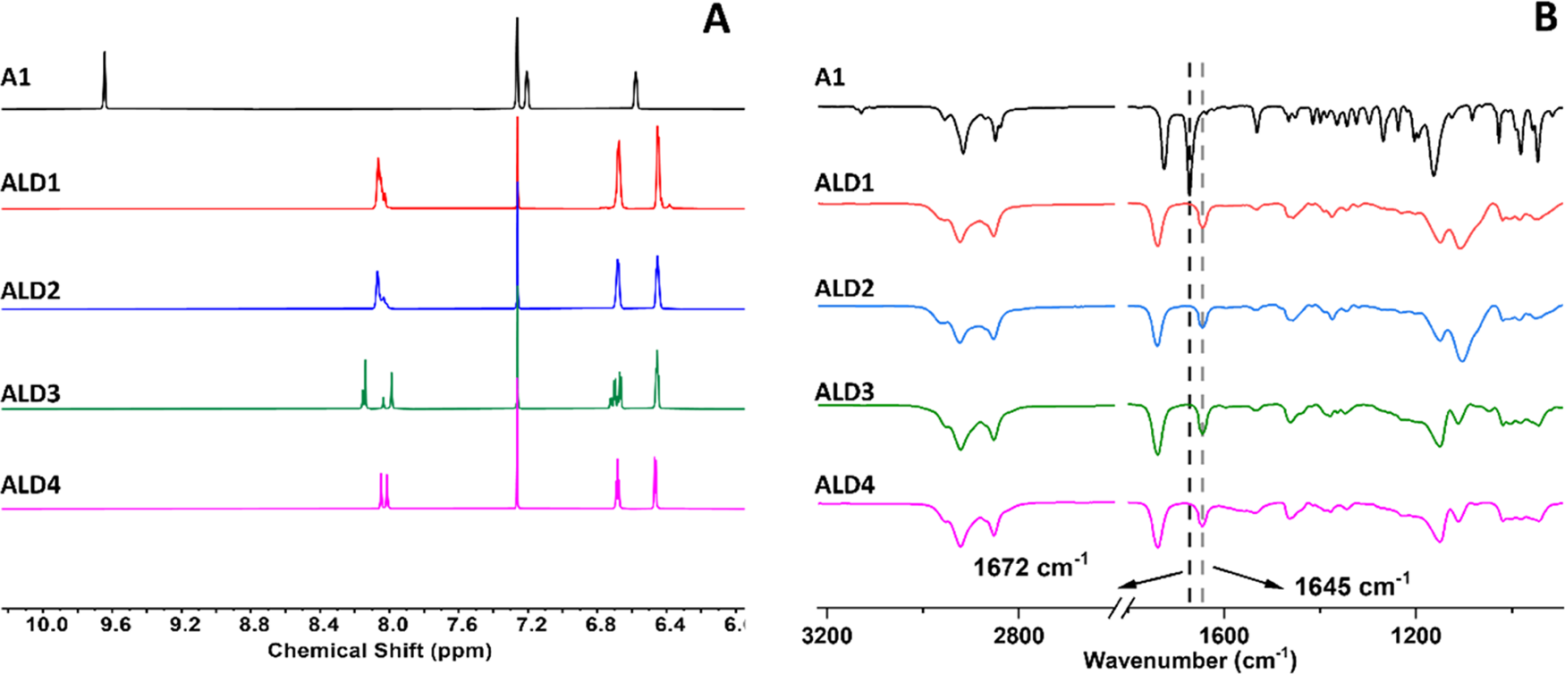
Characterization of aldehyde **A1** and aldimines **ALD1–4** by ^1^H NMR in CDCl_3_ (A) and FTIR (B).

The synthesis of aldehyde **A1**, featuring a C12-ester group, entailed a direct acylation reaction between HMF and lauroyl chloride at room temperature, with pyridine serving as the base. The aldimines were obtained by the straightforward condensation of **A1** with the corresponding amines without further purification (Scheme 1). The structures of **A1** and the aldimines (**ALD1**–**ALD4**) were confirmed by ^1^H and ^13^C NMR and FTIR (Figures 2 and S1–S7). Aldimine formation was determined as the disappearance of the aldehydic proton and carbonyl stretching vibrations at ∼9.6 ppm and ∼1670 cm^–1^ and the emerging imine proton and carbonyl stretching vibration at ∼8.2 ppm and ∼1645 cm^–1^, respectively. The aldimines **ALD1–3** were liquid, whereas **ALD4** was a low-melting solid.

The isocyanate prepolymer was synthesized from a mixture of a diol and triol poly(propylene oxide) polyol and a molar excess of 4,4′-MDI. The prepolymers were synthesized in bulk in the presence of either **A1** or **ALD1–4**, respectively, and subsequently characterized using FTIR analyses (Figures S8–S13). The spectra show the isocyanate stretching vibration (N=C=O) at about 2270 cm^–1^ and a urethane carbonyl (C=O) peak at about 1730 cm^–1^.^40^ The stability of the prepolymer and aldimines during the prepolymerization reaction was evident as there was no discernible appearance of the aldehyde carbonyl stretching peak (C=O) at ∼1680 cm^–1^, and the viscosity of the prepolymer did not change. In contrast, prepolymers that did not contain aldehyde or aldimine were not stable; they turned into a physical gel during their preparation (Figure S14). The gel formation was ascribed to urethane–urethane H-bond formation in the prepolymer. The addition of the aldehyde or aldimine prevented the H-bond formation, likely because the polar functional groups (e.g., ether, ester, imine) of the additives competed with the urethane–urethane H-bonding process.

### Catalysis

**Figure 3:**
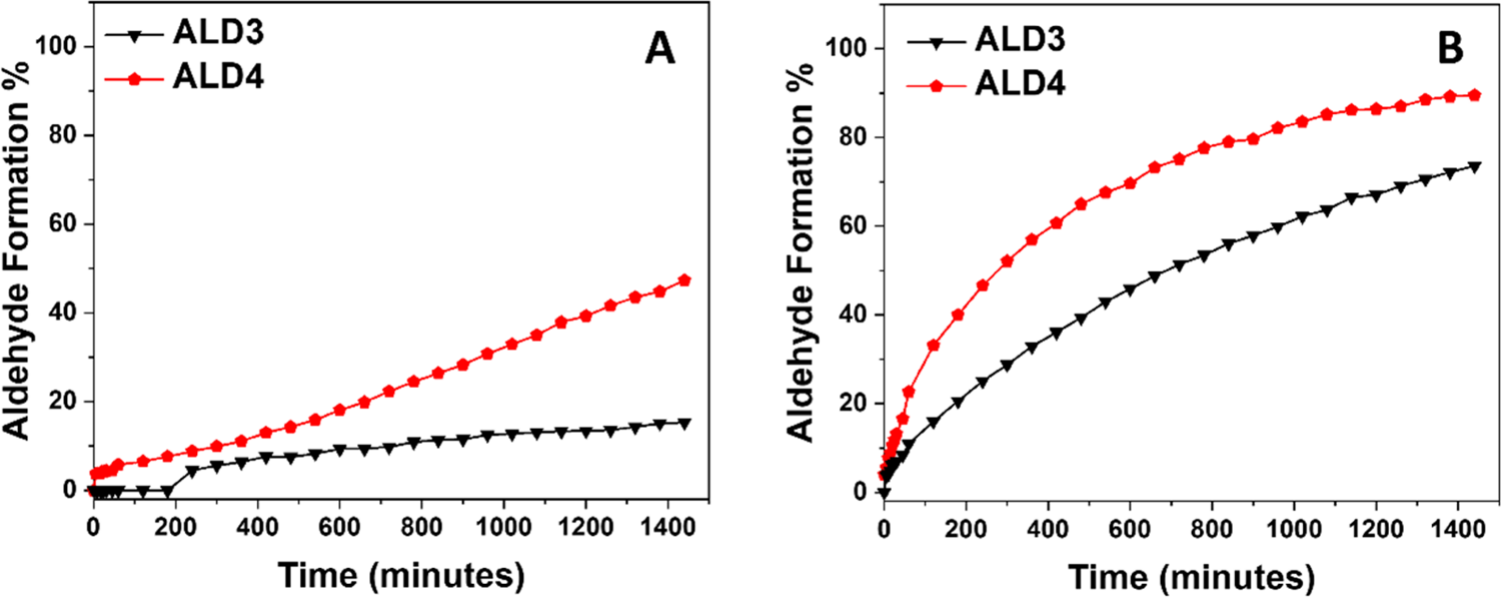
Aldehyde formation with time determined by ^1^H NMR: uncatalyzed system (A), and benzoic acid catalyzed system (B). The conversion was determined from the ratio of the integrals of imine proton vs aldehyde proton (Supporting Information, Figures S15–S18).

To obtain a basic understanding of the curing process, the hydrolysis reaction of aldimine was studied. Hydrolysis experiments were conducted in solution for **ALD3** and **ALD4**. ^1^H NMR was used to follow the progress of the hydrolysis reactions (Figure 3). The reactions were studied in the presence and absence of benzoic acid.

The rate-determining step in the urea reaction from aldimines, water, and isocyanate is the hydrolysis reaction. When the hydrolysis of aldimines is acid-catalyzed, this leads to the protonation of aldimine nitrogen atom, which increases the hydrolysis rate.^41,42^**ALD4** displayed the faster hydrolysis rate, with a half-life of 1550 min in the absence and 270 min in the presence of benzoic acid. On the other hand, **ALD3** exhibited a significantly slower conversion rate, both catalyzed and uncatalyzed; the hydrolysis half-life time with and without catalysis amounted to 5500 and 690 min, respectively (Figures S15–S18). In addition, the slow hydrolysis rate of **ALD3** was ascribed to steric hindrance shielding the aldimine groups for the reaction with water. The slow hydrolysis rate appeared to be fundamental to the long tack-free time (*t*_tf_) and the poor mechanical properties of **PP3** (vide infra, Table 1).

**Table 1:** Preparation Characteristics and Mechanical Properties of the Cured Networks^a^

	*t*_tf_ (min)	bubbles	odor	*E*_t_ (MPa)	σ_m_ (MPa)	ε_b_ (%)	σ_ls_ (MPa)
**PPR1**	240	yes	no	nm	nm	nm	0.6 ± 0.1
**PPR2**	40	no	yes	1.9 ± 0.1	6.0 ± 1.8	253 ± 49	1.0 ± 0.1
**PP1**	60	no	no	1.3 ± 0.1	9.8 ± 0.9	431 ± 29	1.4 ± 0.1
**PP2**	45	no	no	2.1 ± 0.1	2.8 ± 0.6	130 ± 24	1.4 ± 0.2
**PP3**	100	no	no	1.0 ± 0.1	7.8 ± 0.6	446 ± 19	0.9 ± 0.1
**PP4**	15	no	no	1.4 ± 0.1	6.9 ± 1.4	314 ± 34	1.4 ± 0.1
PP1*	80	no	no	1.2 ± 0.1	11.9 ± 0.7	503 ± 54	1.0 ± 0.1
PP2*	55	no	no	1.3 ± 0.1	9.9 ± 0.7	419 ± 22	1.0 ± 0.1

a *t*_tf_: tack-free time, *E*_t_: Young modulus, σ_m_: ultimate true stress, ε_b_: elongation at break, σ_ls_: lap-shear strength, nm: not measured due to bubbles. The asterisk * denotes the systems w/o catalyst.

Moreover, the reaction between water and polyaldimines is preferred over the reaction between water and isocyanates. To substantiate this, we investigated the reaction of *p*-tolylisocyanate with water in the absence of aldimines using ^1^H NMR over a time period of 24 h. Our findings demonstrated an exceedingly prolonged half-life for urea formation (336 h, 20,160 min). This observation suggests that regardless of the presence of benzoic acid catalysis, the hydrolysis of aldimines, along with subsequent chain extension and cross-linking, surpasses the reactivity of *p*-tolylisocyanate with water (Figures S19–S21).

Benzoic acid catalysis was utilized throughout the prepolymer study. The prepolymers, **PP1–PP4**, and the reference prepolymer **PPR2** containing **ALDR** (Incozol BH) contained 20 mol % benzoic acid on aldimine. The reference prepolymer reaction **PPR1** was studied without benzoic acid because this reaction would not benefit from acid addition. PP1* and PP2*, both devoid of acid, were included for reference.

### Preparation of the Castings

The primary cure mechanism in the 1K systems is the reaction of isocyanate and water to form urea and carbon dioxide. The rate of curing relies on the availability of water. The curing process takes place from the outside inward and is controlled by the diffusion rate of water through the curing polymer. Especially in thicker films, the carbon dioxide formed may not be able to diffuse out fast enough, which might cause defects such as pinholes and blisters. The aldimine hydrolysis reaction competes with the water–isocyanate reaction, the extent of which is dependent on the hydrolysis rate of the aldimine. When the added amount of aldimine is high enough and its rate of hydrolysis is significantly faster than that of the isocyanate–water reaction, blister formation may be prohibited.

The quantity of aldimine added to the prepolymer was calculated such that upon a fast hydrolysis of the aldimine and subsequent reaction of the amines with isocyanate the generated polyamine would consume 50% of the originally available isocyanate groups. The remaining isocyanate was reacted with water to form urea and carbon dioxide. In this scenario, the desired outcome is urea formation through the reaction of aldimine, water, and isocyanate outpacing the urea formation of the isocyanate–water reaction. The formation rate of carbon dioxide, especially at the beginning of the curing reaction, would be minimized, reducing the probability of blister formation. However, if the hydrolysis rate of the aldimine decreases to a level comparable to that of the isocyanate–water reaction, its effectiveness in minimizing blisters may diminish, potentially resulting in incomplete network formation. The acid-catalyzed prepolymers **PP1**–**PP4** and **PPR2** showed tack-free times (*t*_tf_) ranging from 15 min (**PP4**) to 100 min (**PP3**) (Table 1). *t*_tf_ for control prepolymer **PPR1** was significantly longer and amounted to 240 min. The uncatalyzed prepolymers PP1*–PP2* (*t*_tf_ = 80 and 55 min, respectively) reacted more slowly than their catalyzed counterparts **PP1**–**PP2** (*t*_tf_ = 60 and 45 min, respectively) but were still faster than **PPR1**. Hence, the addition of aldimine, with and without an acid catalyst, sped up the reaction. This implies that the hydrolysis rate of all of the aldimines and their subsequent reaction with isocyanate was faster than the water–isocyanate reaction, a prerequisite for blister reduction.

The polyether amine precursor in **ALD1–2** and **ALDR** possesses sterically hindered amine groups. **ALD1** and **ALDR** are produced from a two-functional amine, whereas **ALD2** is based on a three-functional amine. The faster curing of **PP2** can be ascribed to the higher functionality of the aldimine, which enhances molecular mass buildup and reduces the conversion of gelation. **ALD1** and **ALDR** are based on aldehyde **A1** and benzaldehyde, respectively. The prepolymer containing **ALDR** reacts faster than that containing **ALD1** (*t*_tf_ = 40 and 60 min, respectively), which suggests that the hydrolysis rate of the benzaldehyde-based aldimine is faster than that of the **A1**-based aldimine.

Two prepolymers stand out, those based on **ALD3** and **ALD4**, being the slowest and fastest, respectively. The aldimine in **ALD3** is based on isophorone diamine, which possesses a sterically hindered and a sterically free amine. Both aldimine groups in **ALD3** are sterically shielded by the substituted isophore spacer moiety. This geometrical constraint reduces the hydrolysis rate of the aldimine and, therefore, the curing of the polymer. **ALD4**, based on 1,5-diamino-2-methylpentane possessing two sterically free amines, undergoes fast hydrolysis. Furthermore, the two urea groups formed in the reaction of the diamine and 4,4′-MDI can form strong intermolecular H-bonding.^43^ The physical association of the diurea segments is an effective mechanism for speeding up the curing and occurs only with **ALD4**.

**Figure 4:**
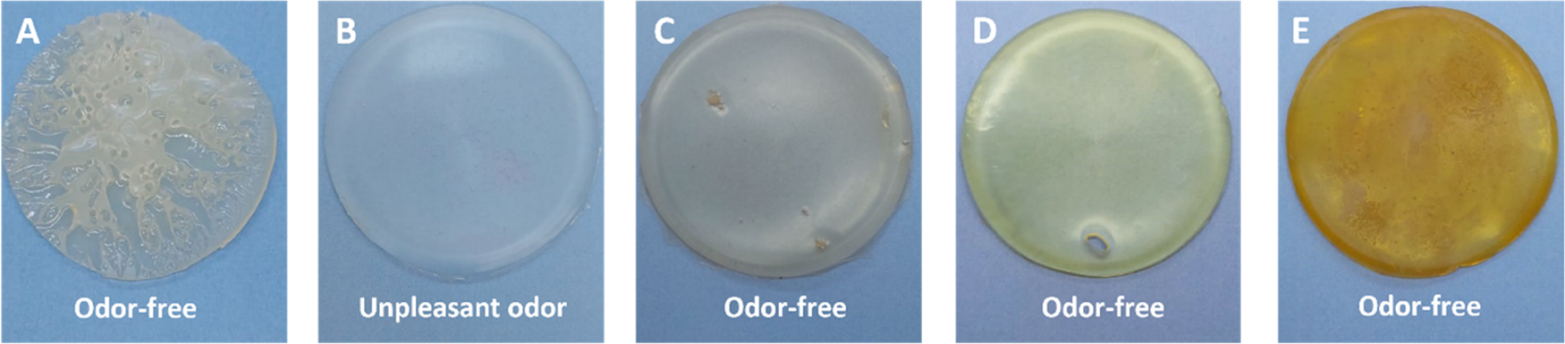
Visual appearance of the selected prepolymers after 1 week of curing at 60% RH: **PPR1** (A), **PPR2** cured with benzoic acid catalyst (B), **PP1** cured without benzoic acid catalyst (C), **PP2** cured with benzoic acid catalyst (D), and **PP4** cured with benzoic acid catalyst (E). A complete series of photographs can be found in Table S2. The color of the castings primarily stems from the inherent brown color of the HMF-based aldimines, which holds little relevance in most adhesive applications.

After 1 week of curing at 60% relative humidity, the curing had finished as evidenced by the complete disappearance of the NCO stretching peak at 2270 cm^–1^ in the FTIR spectra (Figures S8–S13). As expected, the reference prepolymer **PPR1** showed a strong bubble formation. All catalyzed prepolymers yielded defect- and bubble-free films (Figure 4 and Table S2). **PP1** and **PP2**, however, did not require acid catalysis to produce bubble-free films.

The casting produced from **PPR2** containing the benzaldehyde-based aldimine (**ALDR**) had a strong benzaldehyde smell, whereas all of the other castings were essentially odor-free. Benzaldehyde is relatively volatile, and its formation during cure causes smell, a known problem of Incozol BH in the PU industry. **A1** with a molar mass of 308.42 g/mol possesses a sufficiently low vapor pressure and does not emit a smell when formed.

### Mechanical Properties

The castings, based on poly(propylene oxide)-based polyols, 4,4′-MDI, and amine chain extender are amorphous and exhibit an overall low cross-link density. The cross-links in the network originate from the three-functional polyol molecule in the prepolymer. The polymer networks may contain defects that reduce the network density. Such defects originate from monofunctional reactants yielding dangling chains.^1^ Plasticizers may act as a diluent and affect the polymer’s hardness. The occurrence of potential network imperfections and plasticization must be considered when discussing the mechanical properties. Furthermore, when the aldimine is only partially hydrolyzed, this will also lead to the formation of dangling chain ends. The castings prepared from the novel aldimines **ALD1–4** contain **A1** which potentially could act as a plasticizer.

**Figure 5:**
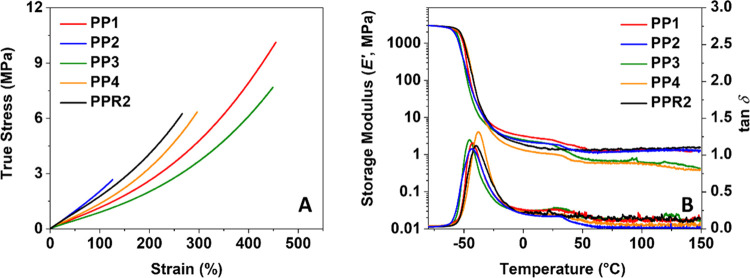
Mechanical characterization of the as-produced castings by tensile testing (A) and DMA (B). More details can be found in Figures S22–S31.

The mechanical performance of the castings was assessed using tensile testing (Figure 5A, Table 1, and Figures S22–S26). The castings are soft and elastic; their Young’s moduli (*E*_t_) and elongation at break (ε_b_) vary between 1 and 2 MPa and 130 and 500%, respectively. The increase in elongation at break and Young’s modulus results in the observed increase in the tensile strength at break. *E*_t_ and ε_b_ are expected to correlate with the cross-link density of the polymer in that *E*_t_ increases and ε_b_ decreases with increased cross-linking. Considering the measured values of *E*_t_ and ε_b_, the polymer network densities can be qualitatively ranked as **PP2** > **PPR2** > **PP4** ≈ **PP1** > **PP3** ≈ PP2* ≈ PP1*.

The thermomechanical characteristics of the networks were assessed using DMA (Figures 5B and S27–S31). The storage modulus *E*′ of 3000 MPa below the glass transition, a value that is typical for amorphous glassy polymers, is the same for all five castings, viz., **PP1–4** and **PPR2**. The elastic modulus declines over 3 orders of magnitude during the glass transition. The decrease in this range is mainly attributed to the coordinated movement of the flexible chain segments of the polyol part, which makes up the larger mass fraction in the polymers (Table S1).

The *E*′ of **PP1–4** declines significantly above 35 °C, whereas **PPR2** does not show softening. The softening in **PP1–4** was attributed to the presence of **A1** (vide infra), the nonvolatile hydrolysis product of **ALD1–4** that stays behind in the polymer. Benzaldehyde, formed in **PPR2** during the hydrolysis reaction of **ALDR**, was volatile and evaporated from the casting (causing the smell). **A1** is a hydrophobic and crystalline compound; it exhibits a melting temperature of 55 °C and is only sparingly soluble in poly(propylene oxide)-based polyols.

The values of tan δ_max_ are given in Table 2. The tan δ_max_ of all five castings is larger than 1. This behavior can be observed in soft PUs and indicates that the polymer is either a gel or a covalently cross-linked network that is plasticized. As mentioned in the beginning of this section, plasticization can be brought about by plasticizing additives, dangling chains, or unbound polymer chains.

**Table 2:** DMA Properties, Cross-Link Density, Swelling Ratio, and Gel Fraction of the Extracted (Denoted x) Networks^a^

	tan δ_max_	*T*_g_ (°C)	tan δ_*x*,max_	*T*_gx_ (°C)	*q* (%)	ϕ (%)	*ν*_e,x_ (mol/m^3^)
**PP1**	1.17	–51	1.08	– 47	454	87	165
**PP2**	1.08	– 55	1.06	– 47	313	92	422
**PP3**	1.21	– 54	1.22	– 47	544	85	107
**PP4**	1.32	– 50	1.23	– 49	475	87	150

a tan δ_max_ and *T*_g_: max. loss factor and *T*_g_ before extraction, tan δ_*x*,m_ and *T*_gx_ ibid. after extraction, tan δ_*x*,50_: loss factor at 50 °C after extraction, *q*: swelling ratio, ϕ: gel fraction, and *ν*_e,x_: cross-link density from *q*.

### Swelling Measurements

The castings **PP1–4** were submerged in THF for a week. The sol/gel (ϕ, respectively (1 – ϕ)) content and the degree of swelling (*q*) of the polymer were determined. The sol fraction was analyzed with ^1^H NMR spectroscopy, and the extracted polymers were resubmitted for DMA and FTIR.

The sol content of **PP2** amounted to 8% and those of **PP1**, **PP3**, and **PP4** were 14%. These data are identical with the calculated theoretical amount of **A1** in the polymers assuming complete hydrolysis. Furthermore, ^1^H NMR revealed that the extracts exclusively consisted of **A1** (Figure S32), and FTIR on the extracted polymers showed that the aldehyde carbonyl stretching (C=O) peak had disappeared (Figure S33). These three observations indicate that the hydrolysis reaction after 1 week of reaction was complete and that **A1** was effectively removed from the polymers upon extraction.

**Figure 6:**
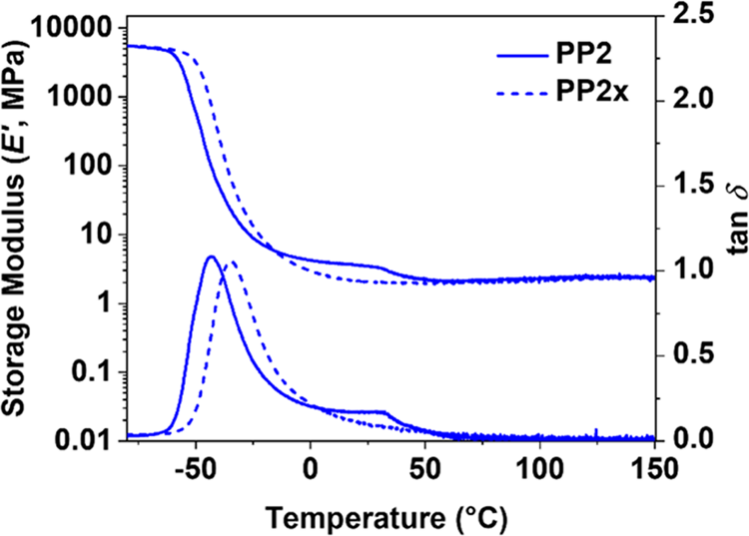
DMA comparison of as-produced and extracted casting **PP2** and **PP2x**.

The comparison of the DMA traces of the as-produced (**PP2**) and extracted (**PP2x**) polymer shows that the minor transition at 35 °C had disappeared after extraction (Figure 6). This proves that the minor transition in DMA at 35 °C of the as-produced castings was caused by the melting of a phase that was rich in **A1**. Extraction, however, also slightly shifted the *E*″ curve to higher temperatures. This shows that part of **A1** was dissolved in the matrix and acted as a plasticizer. Thus, **A1** partially acts as a physical filler (below its melting point) and plasticizer.

The systematic increase in *T*_g_ (defined as the temperature at which *E*″ shows a maximum) upon the extraction of **A1** was observed in all four castings (Table 2 and Figure S34), whereas a decrease in the tan δ_max_ was observed in three of the four castings. Nevertheless, all four extracted polymers showed a tan δ_max_ value larger than 1. This is due to the dangling ends that are present in these polymers, which can be attributed to the unsaturated monofunctional alcohols in the polyols.^1^ The number of elastic chains (*ν*_e_) in the four polymers, calculated from the degree of swelling (*q*), gives the following ranking: **PP2** > **PP1** ≈ **PP4** > **PP3**.

The building blocks of **ALD1** and **ALD2** differ in functionality, but both are produced from polyether amines. The amine groups released after hydrolysis are highly reactive toward isocyanate and chain extension and cross-linking, respectively, occur. The higher amine functionality of **ALD2** explains the higher cross-link density of **PP2**. **ALD4** hydrolyzes readily (Figure 3) and the cross-link density of the corresponding **PP4** is comparable to that of **PP1**. The higher number of dangling ends in **PP3** likely results from the bulkiness and structural asymmetry of the isophorone aldimine moiety in **ALD3**. The aldimine from the sterically free amine will hydrolyze more readily than that from the sterically hindered amine. The generated primary amine will react quickly with isocyanate. It appears, however, that the hydrolysis of the second aldimine is slower than the water–isocyanate reaction, which results in the formation of dangling chains. With time, the sterically more hindered aldimine will hydrolyze, but by then, the isocyanate groups will all have reacted with moisture from the environment.

### Wood Adhesion

When adhesion performance is assessed, it is crucial to examine the failure mode upon the application of force. This is known as cohesive failure when the adhesive material leaves a residue on both surfaces following a failure. Conversely, if the surfaces to which the adhesive was applied remain clean, this is referred to as adhesive failure.^44^ It is imperative for the application that the adhesive exhibits cohesive failure.^1,45^ All 1K systems presented in this work showed cohesive failure, demonstrating that the adhesive forces exceed the cohesive strength of the polymer (Figures S35 and S40) and that the polymer is the weakest link in the chain.

**Figure 7:**
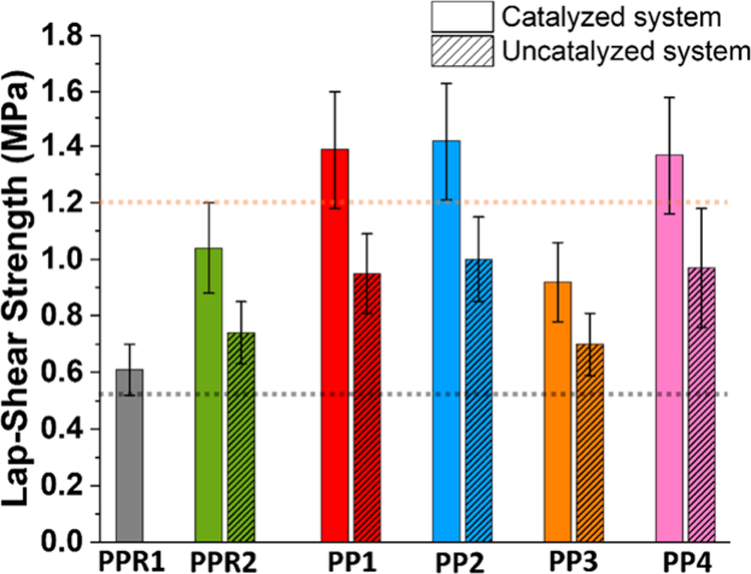
Lap-shear strength: columns w and w/o patterning represent uncatalyzed and acid-catalyzed systems, respectively. Dashed lines are drawn for clarity.

The prepolymer systems were evaluated in a basic wood adhesive application. The lap-shear results (lap-shear curves in Figures S35–S40) are numerically given in Table 1 and visually represented in Figure 7 (experimental error in shear strength 15%). The shear strength of the catalyzed system always exceeded that of the corresponding uncatalyzed system, likely because of the improved strength (i.e., cohesive energy) of the polymer. The lap-shear strength of **PPR2** and **PP3** without acid catalysis was close to that of **PPR1** with 0.6 MPa (black dashed line as the lower bound reference), whereas **PP1**, **PP2**, and **PP4** without acid showed shear strength values of 1 MPa. **PP1**, **PP2**, and **PP4** in the presence of benzoic acid showed the highest shear strength values of about 1.4 MPa (orange dashed line as upper bound reference), whereas acid addition to **PPR2** and **PP3** only increased the shear strength to 1.0 MPa. The poor performance of **PP3** is likely related to poor network formation. The superior polymer properties of **PP2**, however, were not significantly reflected in the shear strength results.

## Conclusions

Novel aldimine additives, derived from biobased hydroxymethyl furfural and multifunctional amines, were synthesized and evaluated as latent curing agents in moisture-curing 1K-polyurethane systems. The aldimine chemistry could effectively compete with the water–isocyanate reaction and thus avoid bubble formation. When applied, the aldimines were hydrolyzed with moisture from the environment to produce the aldehyde and polyamine. Then, the polyamine reacts with the isocyanate and cures the polymer. The high molecular mass of the aldehyde prohibited odor release. Acid catalysis of the aldimine hydrolysis reaction reduced the cure time and improved the mechanical properties of the final networks. The cure rate and mechanical properties could be tuned by the choice of polyamine.

## Supporting Information Available

Additional experimental data including full characterization of the aldehyde and aldimines based on ^1^H NMR, ^13^C NMR, and FTIR; visual appearance of the cured PU networks, structural (FTIR), thermal, thermomechanical, and mechanical characterization of the cured PU networks; and lap-shear assessment (PDF)
