# Supplementary material for: Novel Furfural-Derived Polyaldimines as Latent Hardeners for Polyurethane Adhesives
Source: ACS Appl Mater Interfaces. 2024 Jan 29;16(5):6414–23. doi: 10.1021/acsami.3c17416 (PMC10859888; doi:10.1021/acsami.3c17416)
Supplement: Supplementary file 1 — am3c17416_si_001.pdf [file am3c17416_si_001.pdf]

Supporting Information

Novel Furfural-Derived Polyaldimines as Latent Hardeners for Polyurethane Adhesives

Tankut Türel,[a] Berend Eling,[b] Anna M. Cristadoro,[c] Thomas Mathieu,[c] Martin Linnenbrink,[c] Željko Tomović[a,d]**

a. Polymer Performance Materials Group, Department of Chemical Engineering and Chemistry, Eindhoven University of Technology, 5600 MB Eindhoven, The Netherlands.

b. Institute of Technical and Macromolecular Chemistry, University of Hamburg, Bundesstrasse 45, 20146 Hamburg, Germany.

c. BASF Polyurethanes, Elastogranstrasse 60, Lemfoerde, 49448, Germany.

d. Institute for Complex Molecular Systems, Eindhoven University of Technology, 5600 MB Eindhoven, The Netherlands.

Corresponding Authors

Željko Tomović

Polymer Performance Materials Group, Department of Chemical Engineering and Chemistry and Institute for Complex Molecular Systems, Eindhoven University of Technology, 5600MB Eindhoven, The Netherlands.

E-mail: z.tomovic@tue.nl

Martin Linnenbrink – BASF Polyurethanes, Elastogranstrasse 60, Lemfoerde, 49448, Germany.

E-mail: martin.linnenbrink@basf.com

Table of Contents

1) Curing Pathways of Isocyanate Prepolymers S3
2) Characterization of Aldehydes and Aldimines.. S4
3) Synthesis and Characterization of Isocyanate Prepolymers S10
4) Kinetic studies using ^{1}H NMR.. S14
5) Visual Appearance of the Cured Prepolymers S19
6) Tensile Stress-Strain Experiments S20
7) Dynamic Mechanical Analysis S22
8) Analysis of the Extracts & Networks After Extraction S24
9) Dynamic Mechanical Analysis of Extracted Networks.. S25
10) Lap-Shear Tests S26

1) Curing Pathways of Isocyanate Prepolymers

Scheme S1. Curing of isocyanate prepolymer in the absence and in the presence of aldimines. The color coding of the structures are well-aligned with the Figure 1 in the manuscript file.

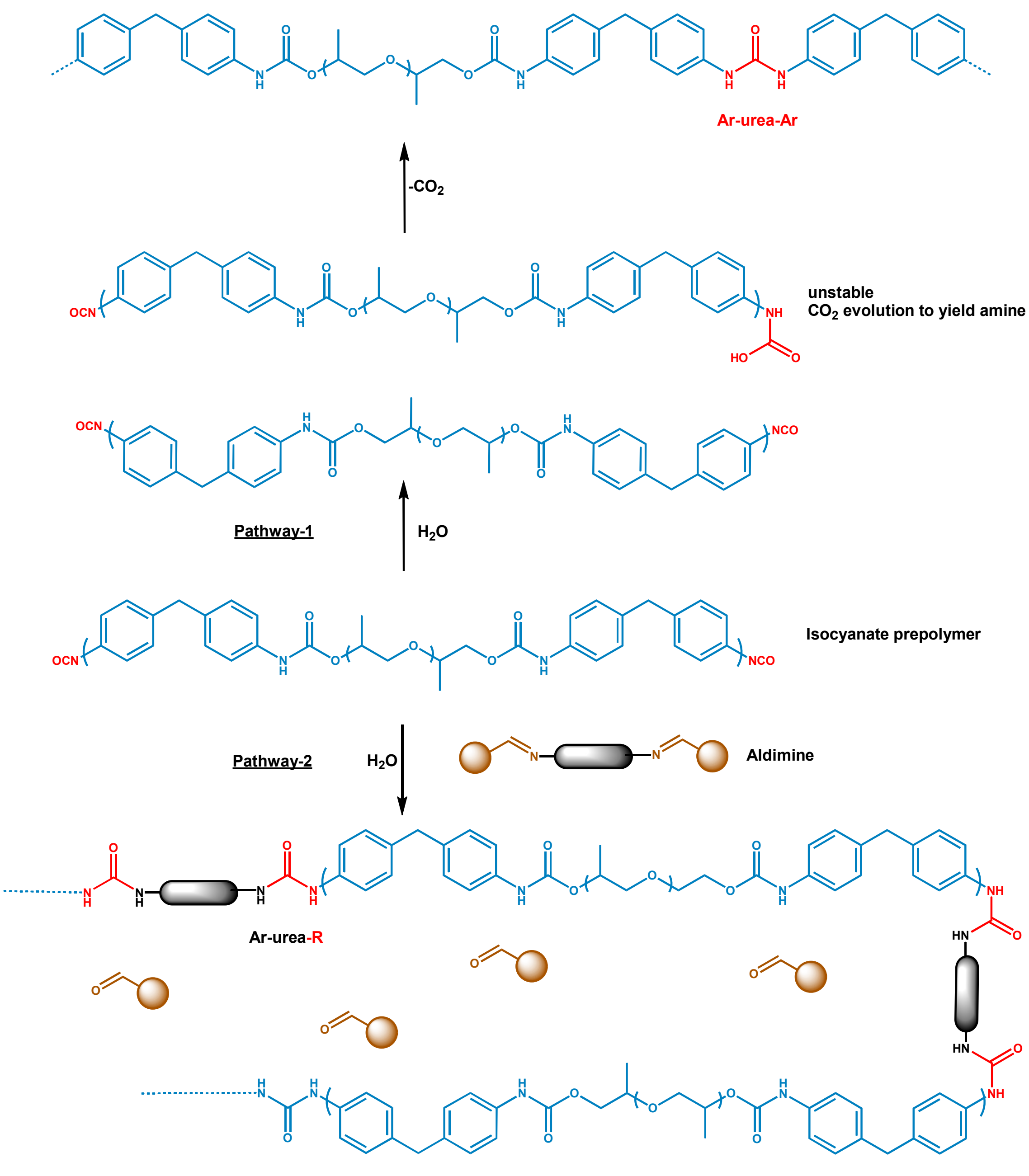

2) Characterization of Aldehydes and Aldimines

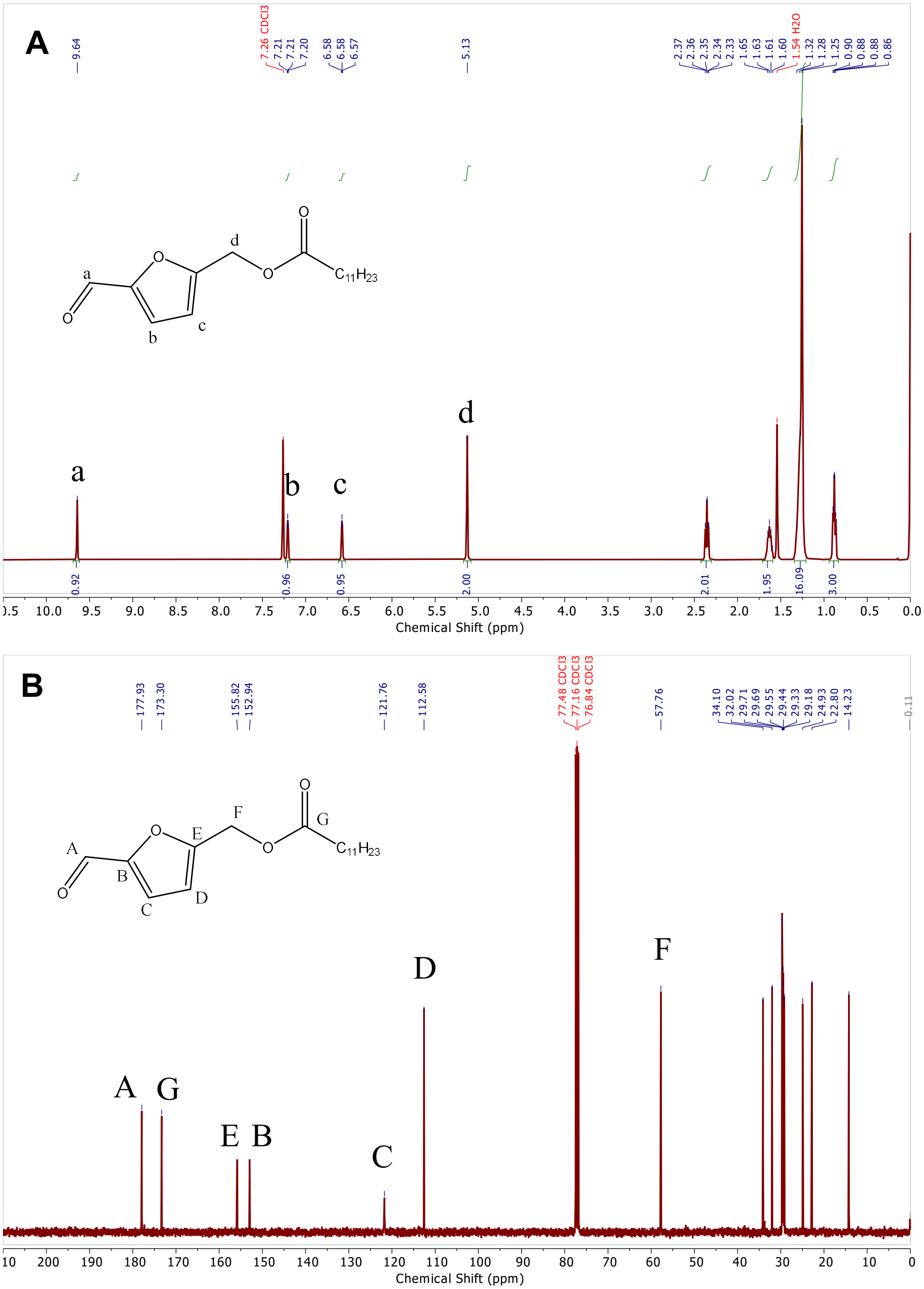

Figure S1. ^{1}H NMR (A) and ^{13}C NMR (B) spectra of **A1** in $CDCl_3$.

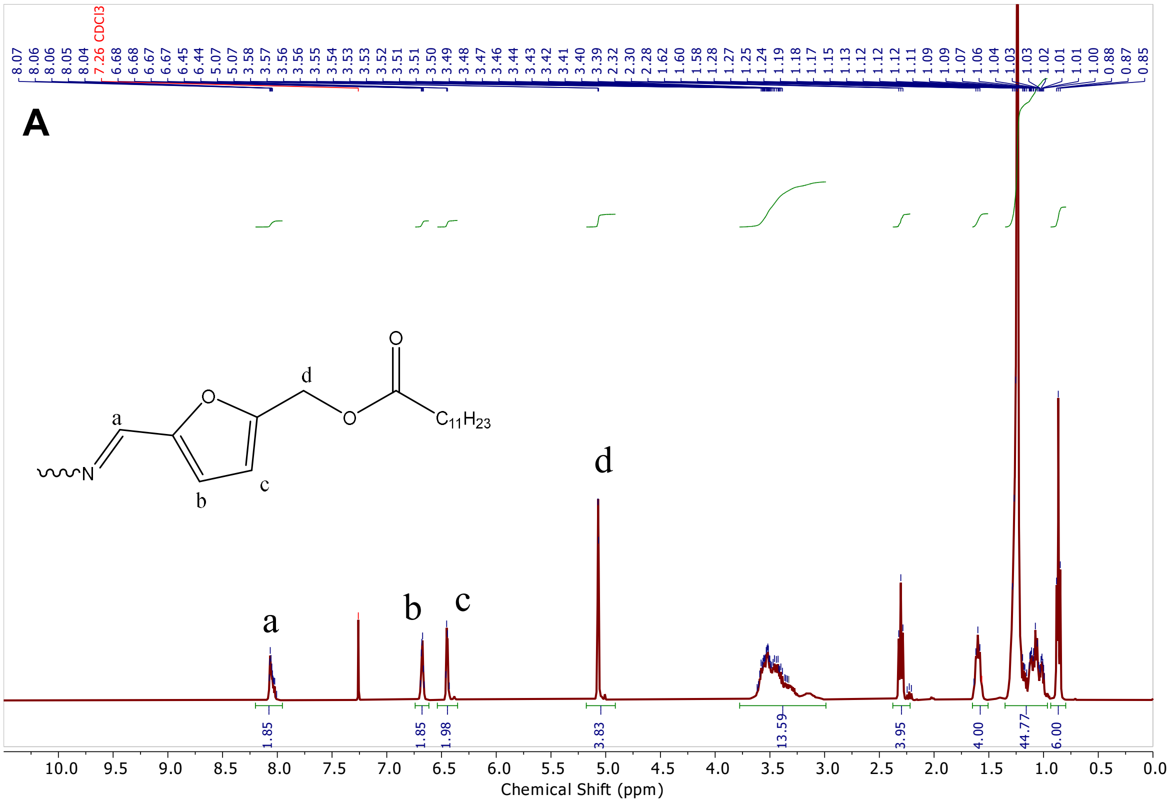

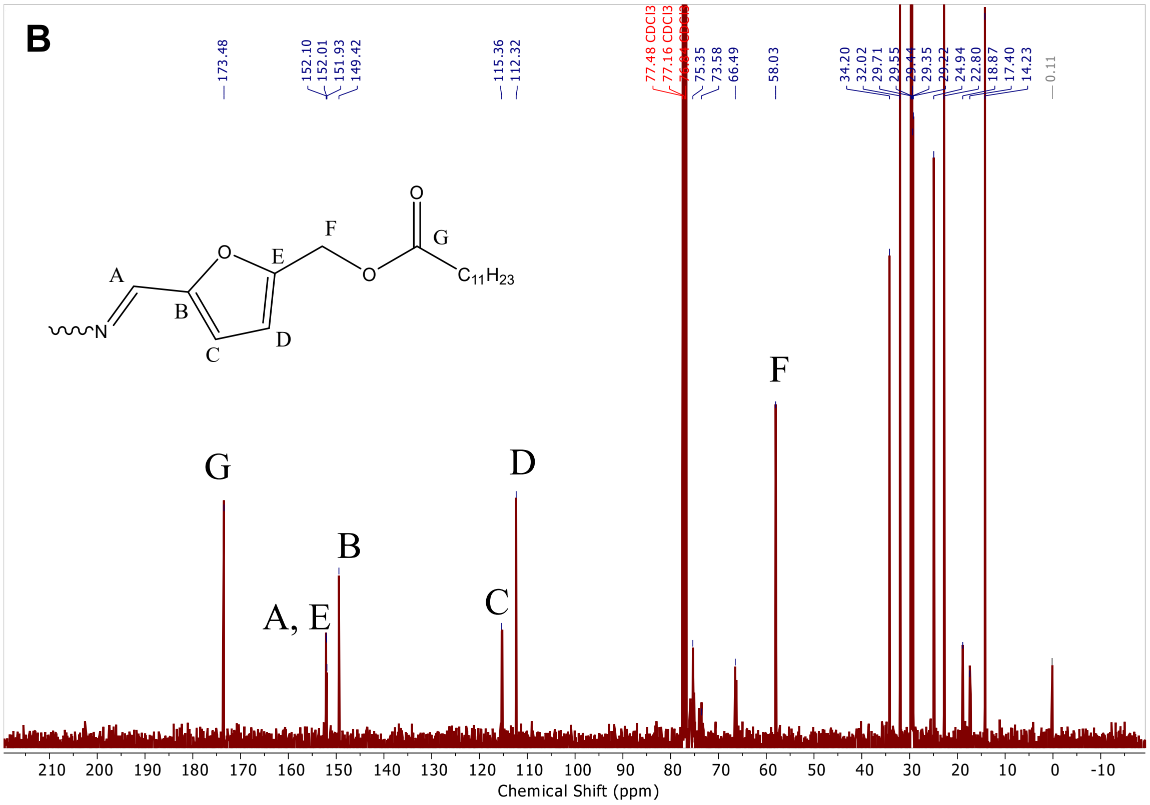

Figure S2. ^{1}H NMR (A) and ^{13}C NMR (B) spectra of **ALD1** in $CDCl_3$.

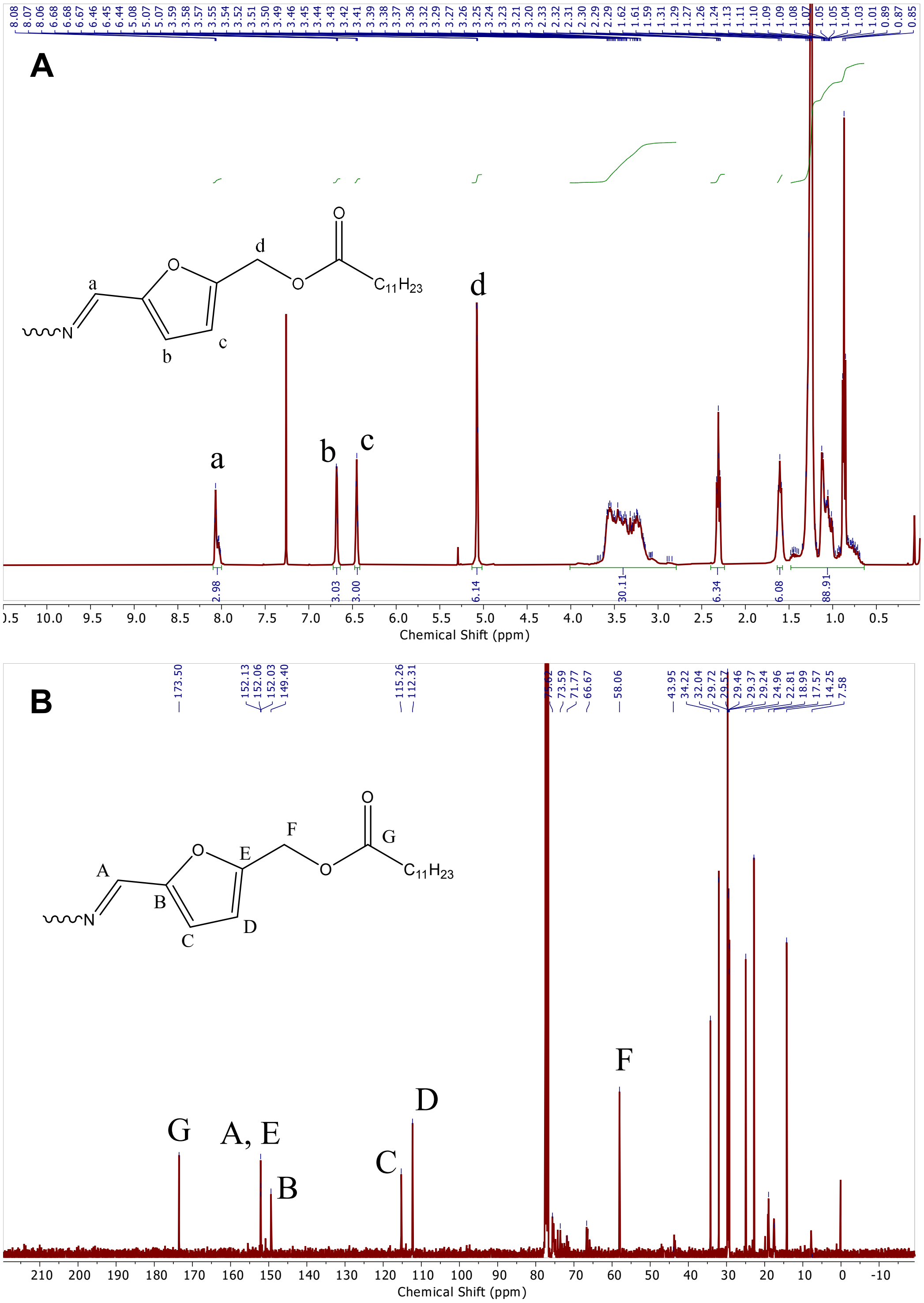

Figure S3. ^{1}H NMR (A) and ^{13}C NMR (B) spectra of **ALD2** in $CDCl_3$.

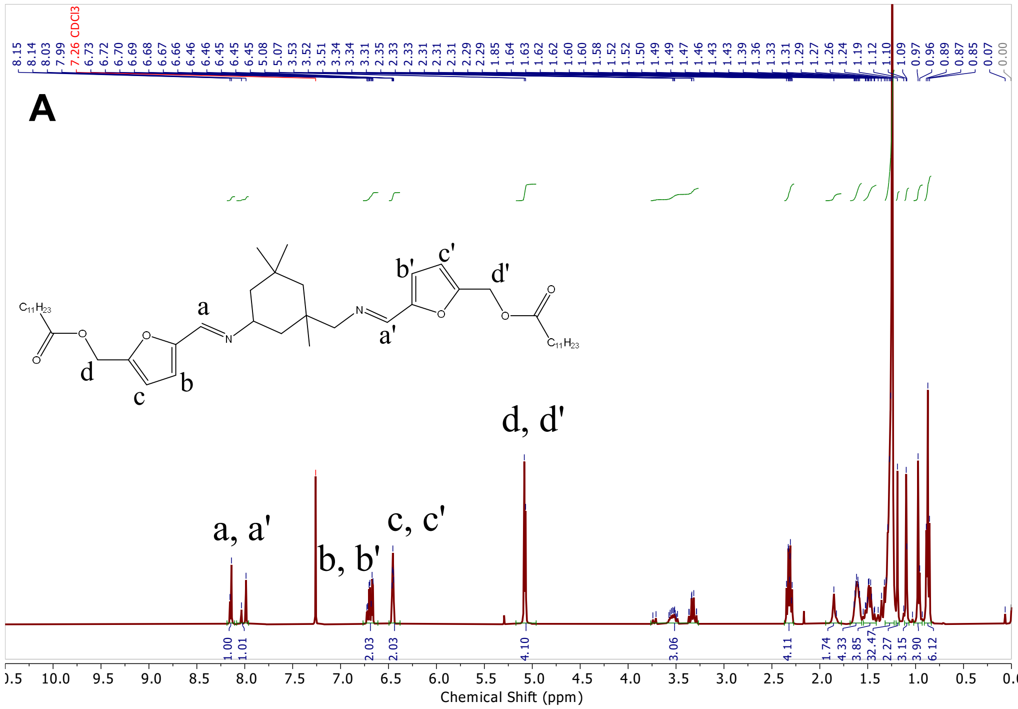

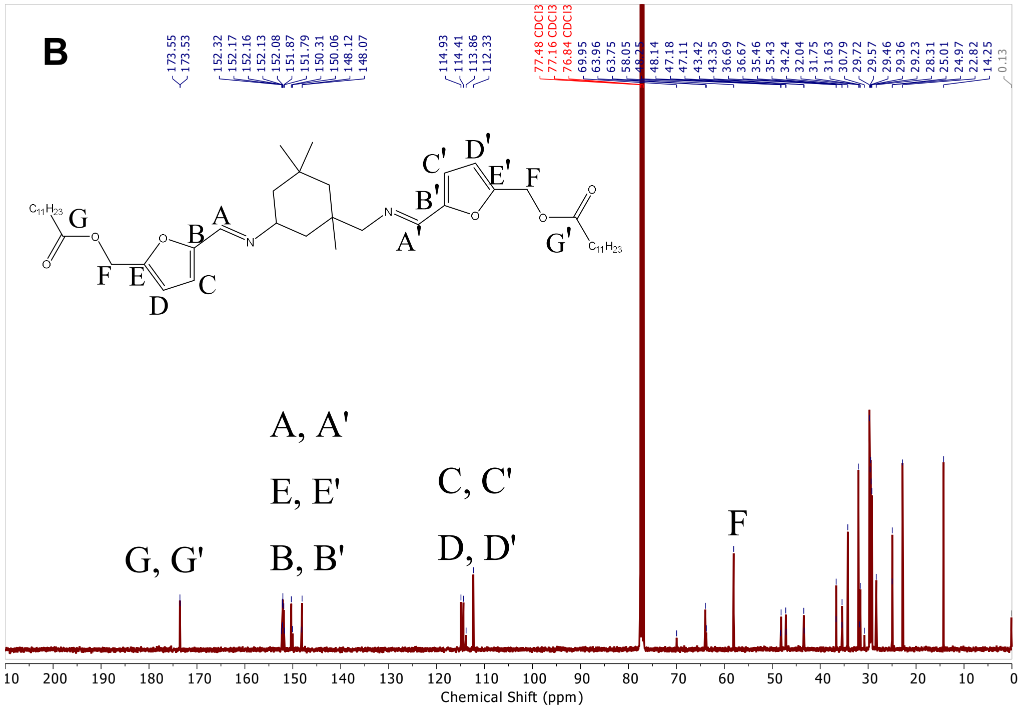

Figure S4. ^{1}H NMR (A) and ^{13}C NMR (B) spectra of **ALD3** in $CDCl_3$. Note that isophorone diamine is not optically pure.

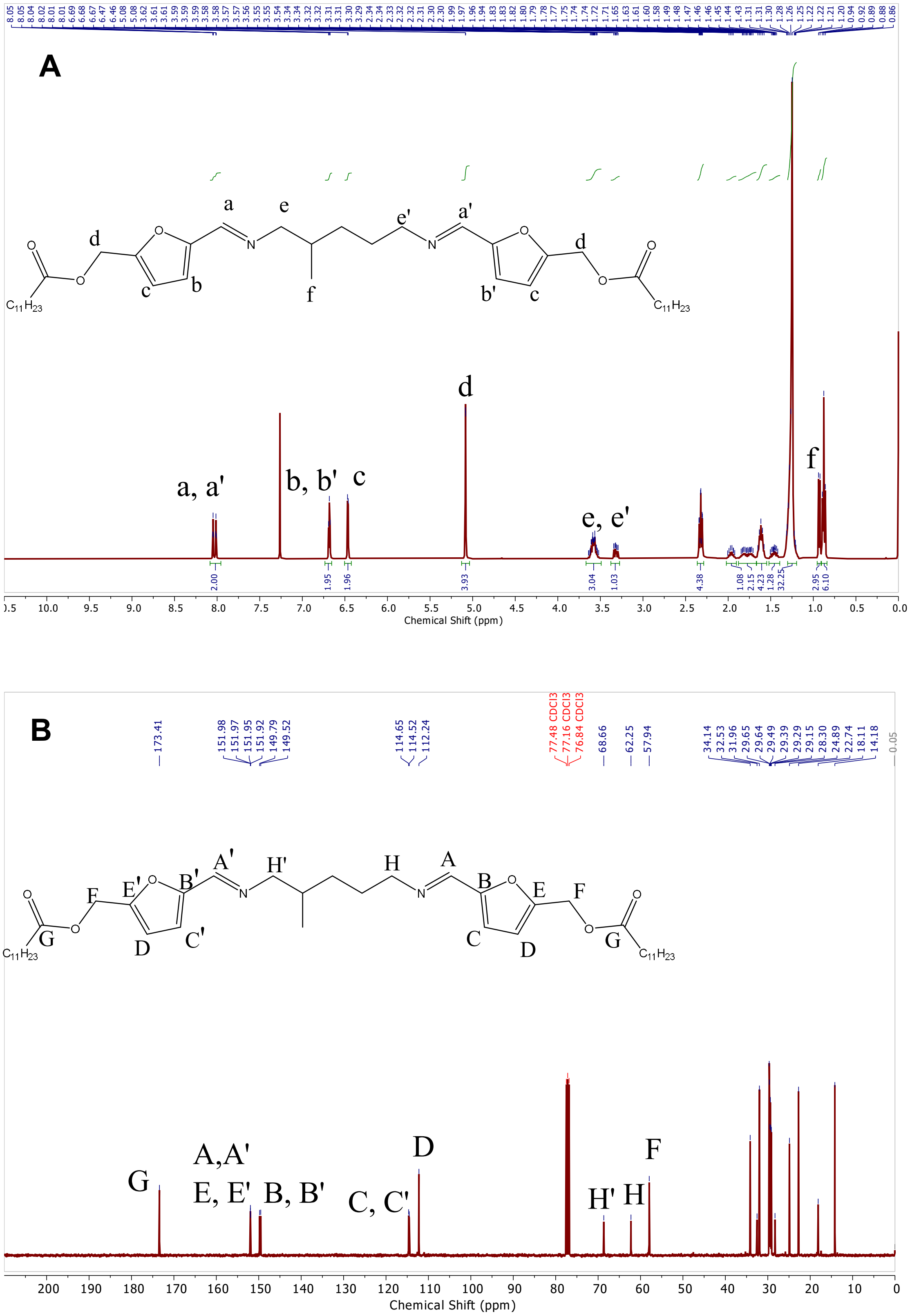

Figure S5. ^{1}H NMR (A) and ^{13}C NMR (B) spectra of **ALD4** in $CDCl_3$. Note that 1,5-Diamino-2-methylpentane is not optically pure.

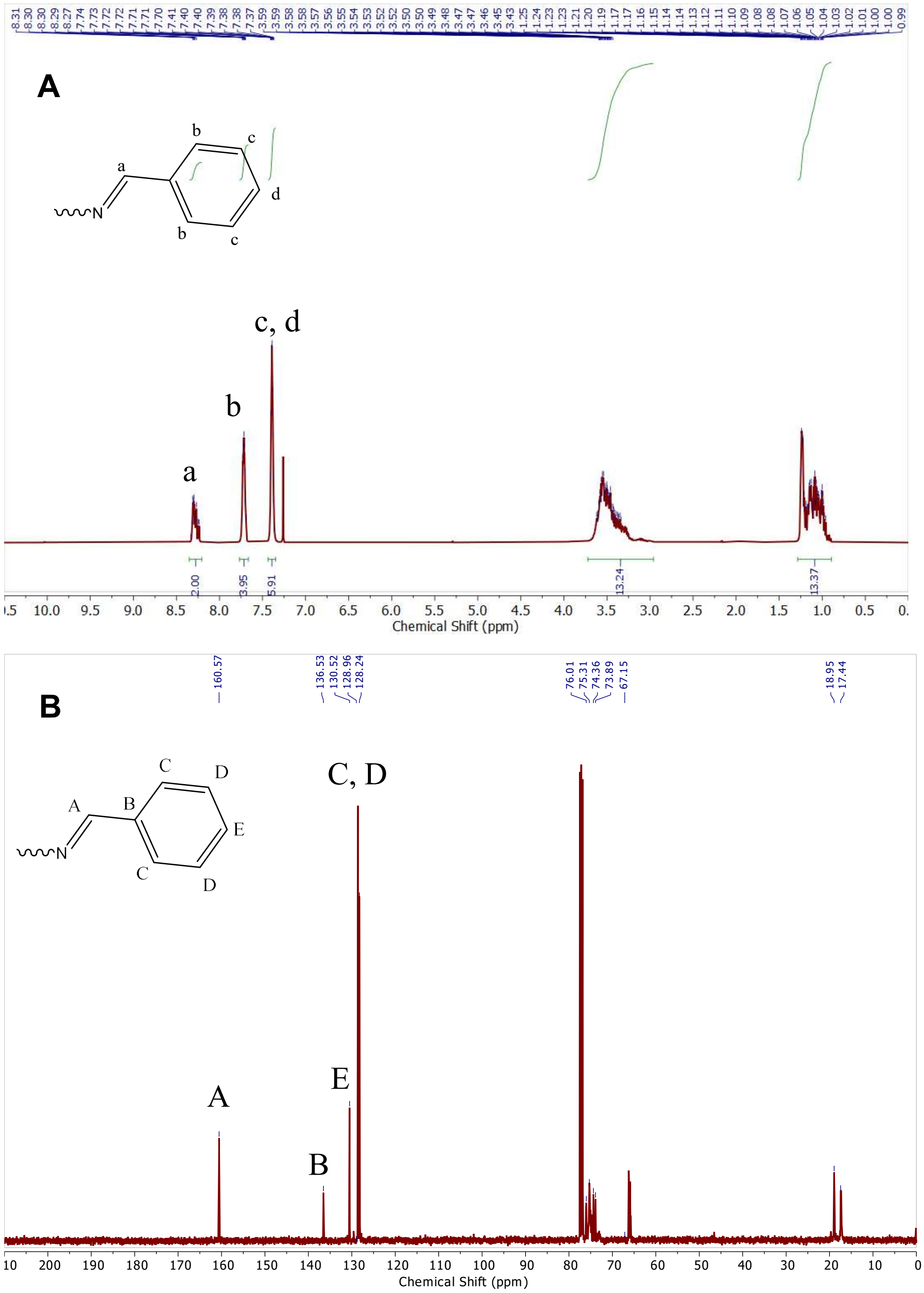

Figure S6. ^{1}H NMR (A) and ^{13}C NMR (B) spectra of **ALDR** in $CDCl_3$.

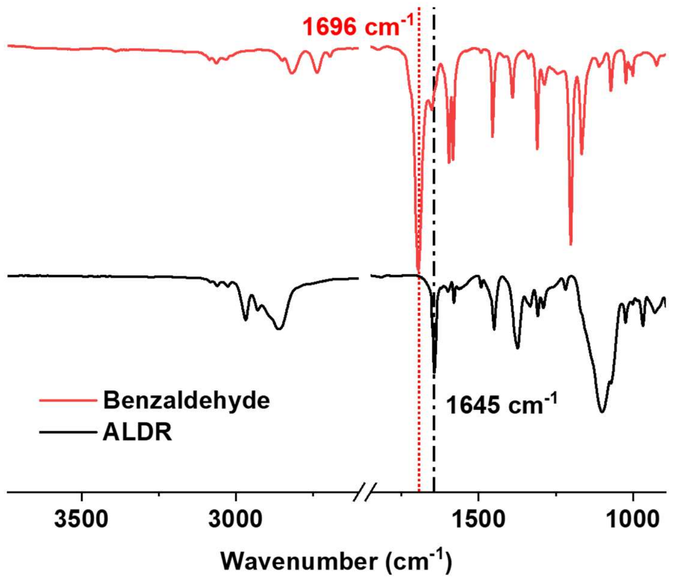

Figure S7. FTIR spectra of **benzaldehyde** (red) and **ALDR** (black).

3) Synthesis and Characterization of Isocyanate Prepolymers

Table S1. Ingredients of the prepolymers.

	PP1	PP2	PP3	PP4	PPR2
**Lupranol 2095**	25.85 g	25.85 g	25.85 g	25.85 g	25.85 g
**Lupranol 1005/1**	12.95 g	12.95 g	12.95 g	12.95 g	12.95 g
**MDI**	6.2 g	6.2 g	6.2 g	6.2 g	6.2 g
**DABCO**	56 mg	56 mg	56 mg	56 mg	56 mg
**ALD1**	5.43 g	-	-	-	-
**ALD2**	-	5.98 g	-	-	-
**ALD3**	-	-	5.05 g	-	-
**ALD4**	-	-	-	4.90 g	-
**ALDR**	-	-	-	-	2.75 g

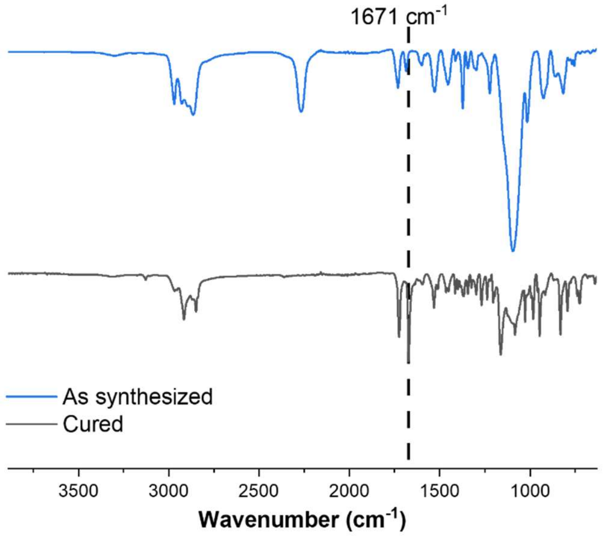

Figure S8. FTIR spectra of reference prepolymer containing aldehyde **A1** (**PPR1**): As synthesized (blue), and after one week of curing under 60% relative humidity (black).

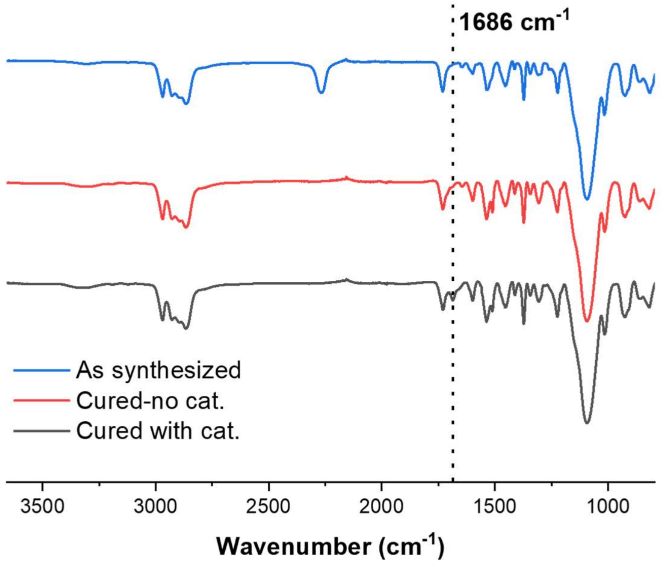

Figure S9. FTIR spectra of prepolymer containing aldimine **ALD1** (**PP1**): As synthesized (blue), after one week of curing under 60% relative humidity without benzoic acid catalysis (red) and after one week of curing under 60% relative humidity in the presence of benzoic acid catalysis (black).

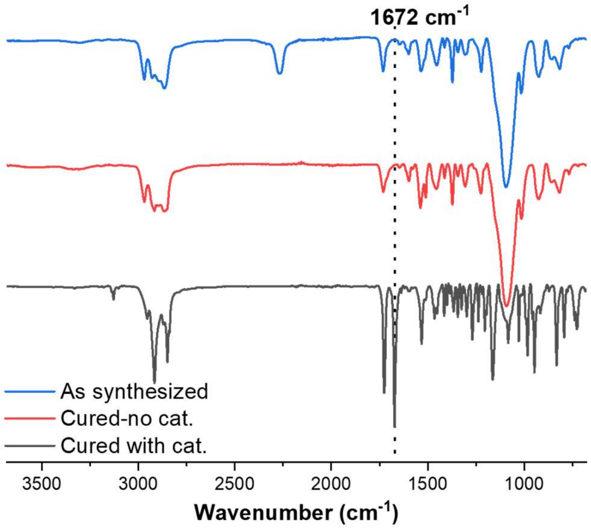

Figure S10. FTIR spectra of prepolymer containing aldimine **ALD2** (**PP2**): As synthesized (blue), after one week of curing under 60% relative humidity without benzoic acid catalysis (red) and after one week of curing under 60% relative humidity in the presence of benzoic acid catalysis (black).

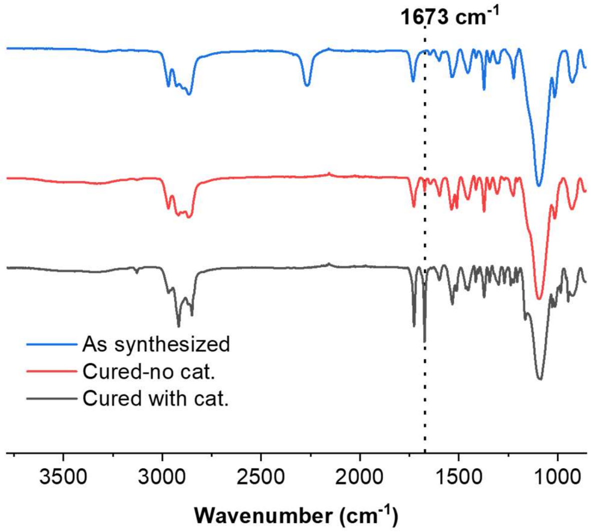

Figure S11. FTIR spectra of prepolymer containing aldimine **ALD3** (**PP3**): As synthesized (blue), after one week of curing under 60% relative humidity without benzoic acid catalysis (red) and after one week of curing under 60% relative humidity in the presence of benzoic acid catalysis (black).

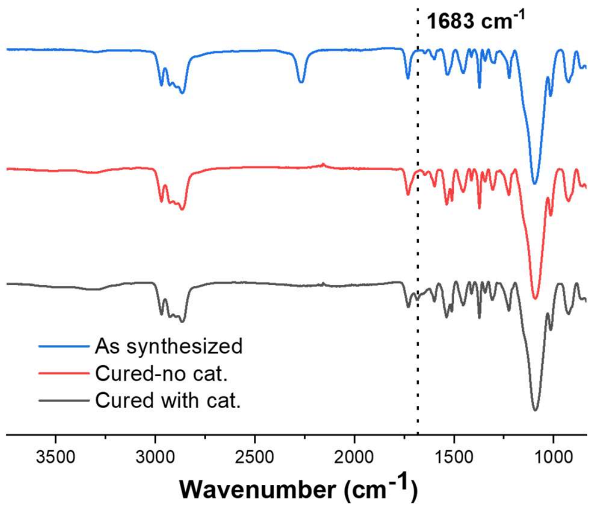

Figure S12. FTIR spectra of prepolymer containing aldimine **ALD4** (**PP4**): As synthesized (blue), after one week of curing under 60% relative humidity without benzoic acid catalysis (red) and after one week of curing under 60% relative humidity in the presence of benzoic acid catalysis (black).

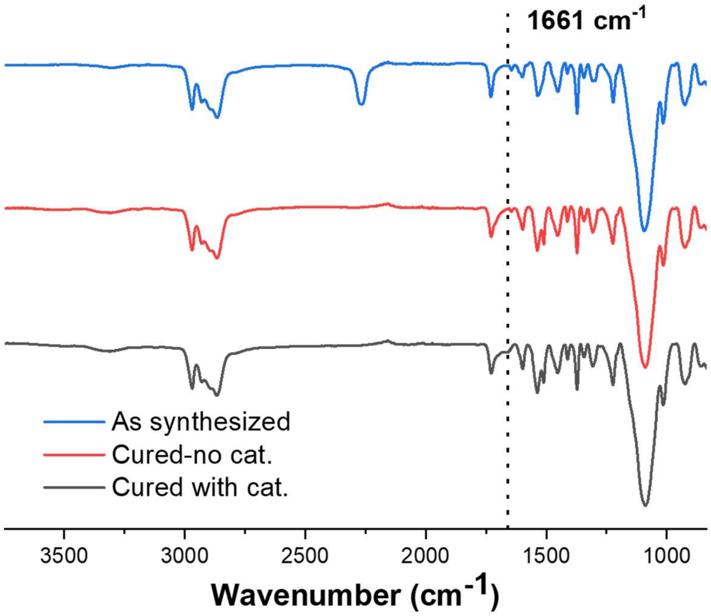

Figure S13. FTIR spectra of prepolymer containing aldimine **ALDR** (**PPR2**): As synthesized (blue), after one week of curing under 60% relative humidity without benzoic acid catalysis (red) and after one week of curing under 60% relative humidity in the presence of benzoic acid catalysis (black).

Ingredients	Amount
Lupranol 1005/1	12.95 g
Lupranol 2095	25.85 g
DABCO	62 mg
MDI	6.25 g
Aldehyde/Aldimine/Plasticizer	0

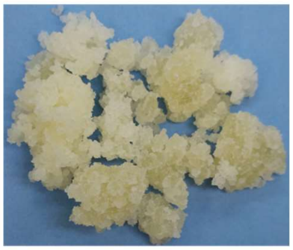

Figure S14. Gelation upon the synthesis trial of isocyanate prepolymer without using **A1** or the aldimines.

4) Kinetic studies using ^{1}H NMR

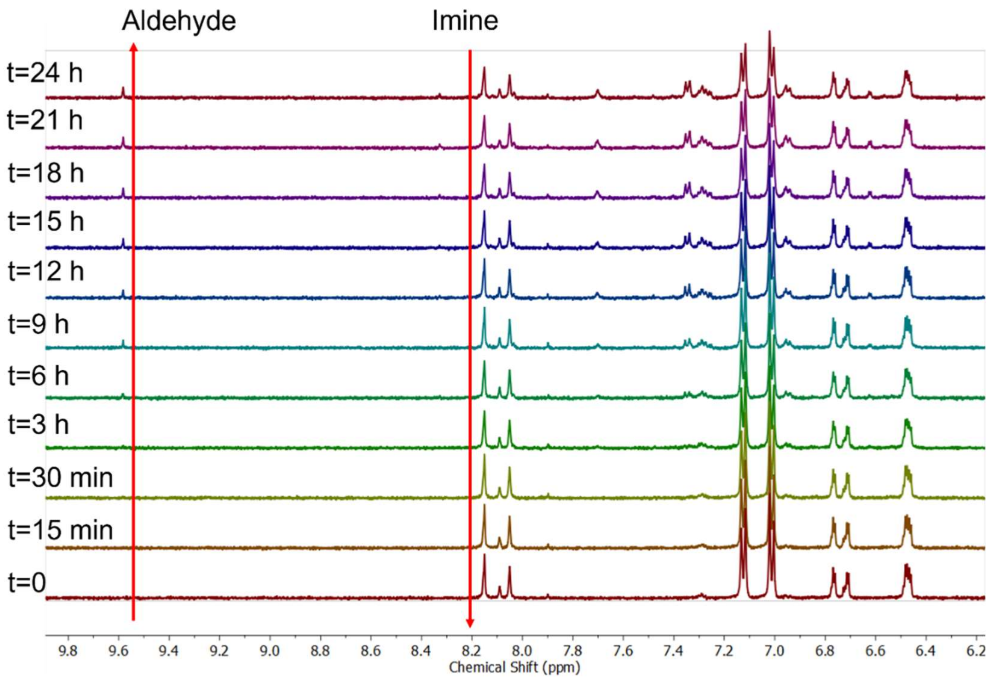

Figure S15. ^{1}H NMR spectra of **ALD3** in THF-d_8 recorded over a 24-hour period in the presence of water and p-tolylisocyanate without catalysis.

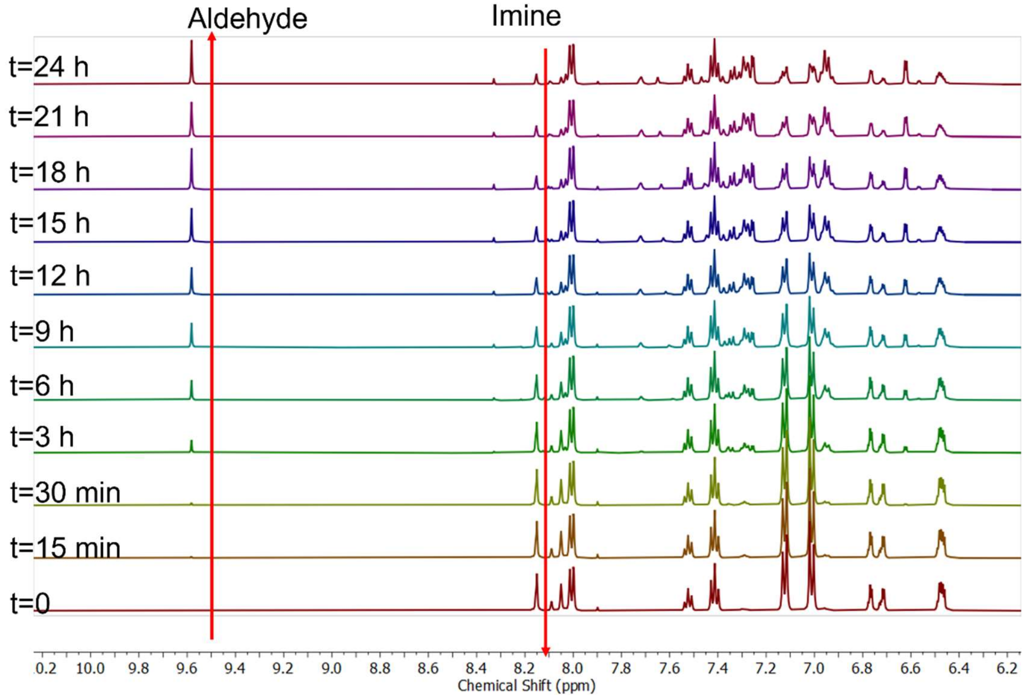

Figure S16. ^{1}H NMR spectra of **ALD3** in THF-d_8 recorded over a 24-hour period in the presence of water and p-tolylisocyanate under benzoic acid catalysis.

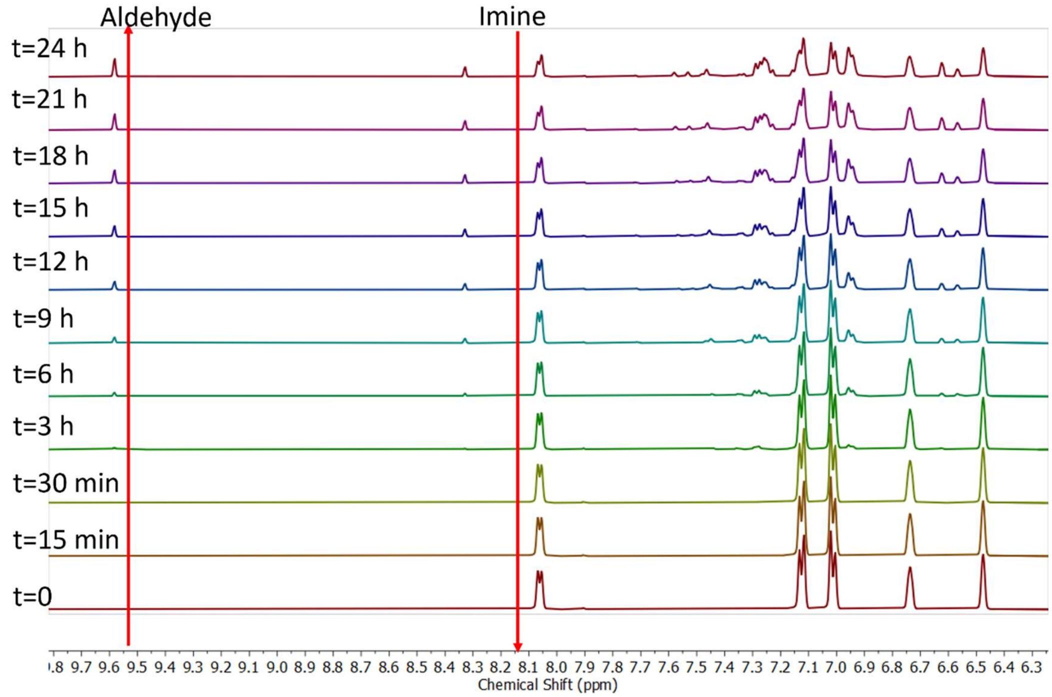

Figure S17. ^{1}H NMR spectra of **ALD4** in THF-d_8 recorded over a 24-hour period in the presence of water and p-tolylisocyanate without catalysis.

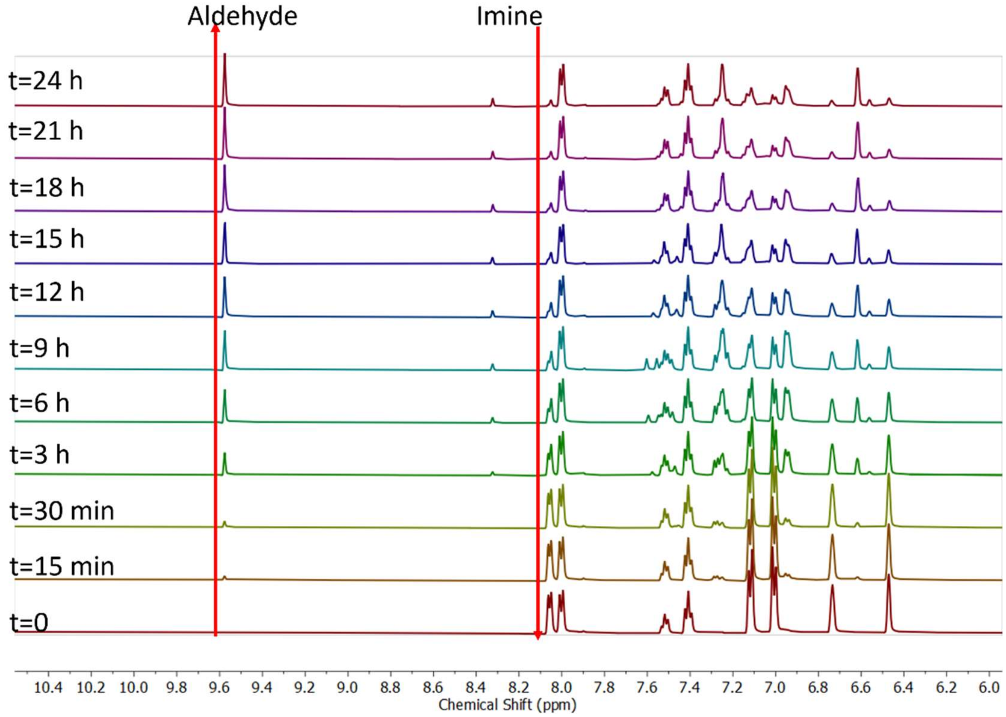

Figure S18. ^{1}H NMR spectra of **ALD4** in THF-d_8 recorded over a 24-hour period in the presence of water and p-tolylisocyanate under benzoic acid catalysis.

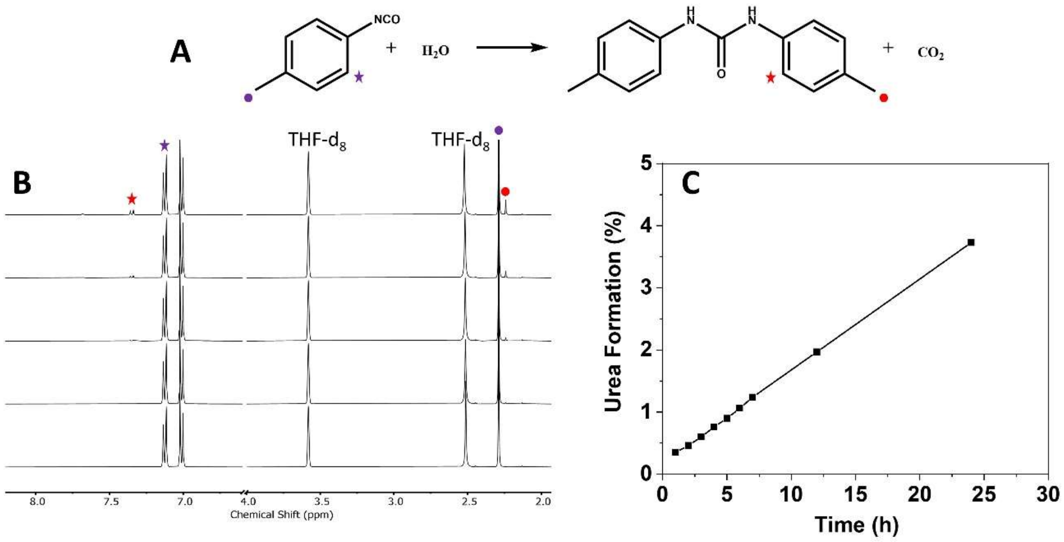

Figure S19. Reaction scheme of p-tolylisocyanate with water to form urea (A), ^{1}H NMR spectra of p-tolylisocyanate in THF-d_8 recorded over a 24-hour period in the presence of water (B), urea formation with time determined by ^{1}H NMR (C). The half-life time for urea formation was determined by extrapolating the linear line to y=50% ($t_{1/2}$=336 h).

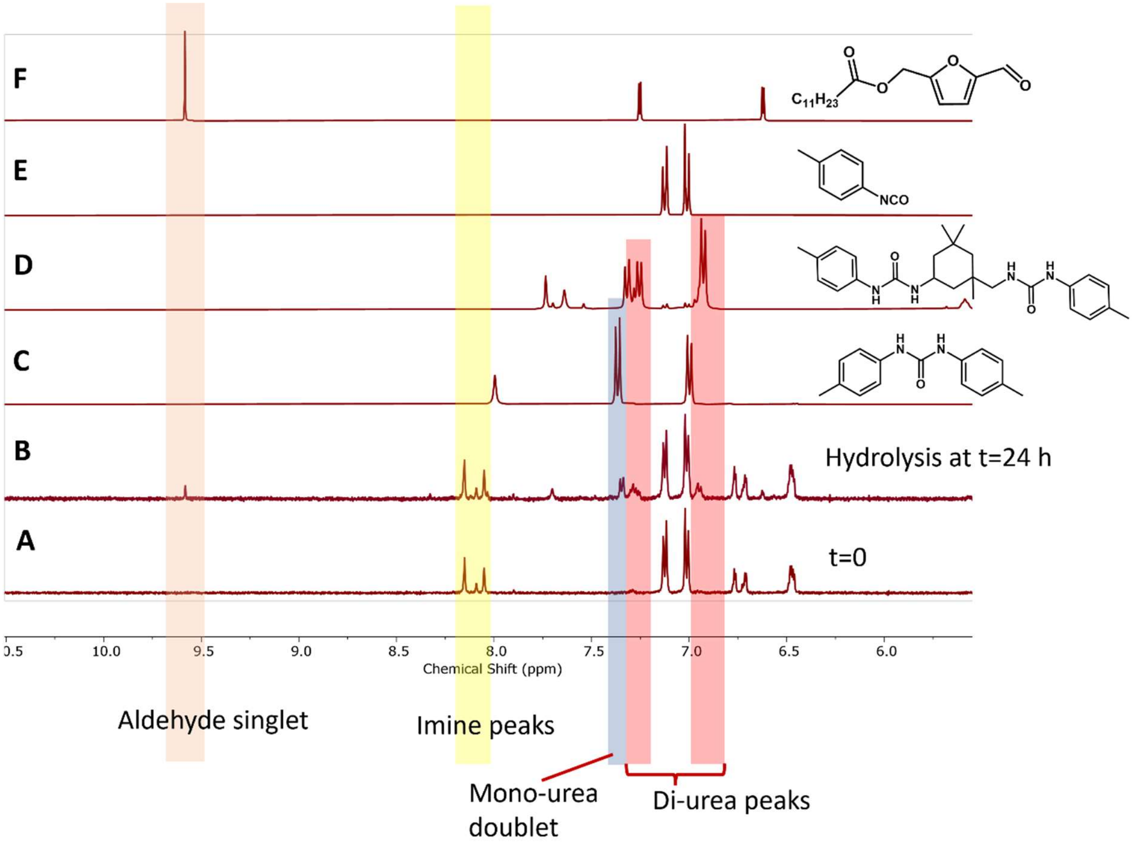

Figure S20. ^{1}H NMR spectra of hydrolysis product of **ALD3** in the presence of water and p-tolylisocyanate without catalysis at t=0 (A) and after 24 h (B), mono-urea compound synthesized through the reaction of p-tolylisocyanate and excess water (C), di-urea compound synthesized through the reaction of p-tolylisocyanate and isophorone diamine (D), p-tolylisocyanate (E), and **A1** (F) recorded in THF-d_8. The ^{1}H NMR comparison shows that di-urea formation (product D) cannot completely outpace the mono-urea formation (product C) without benzoic acid catalysis.

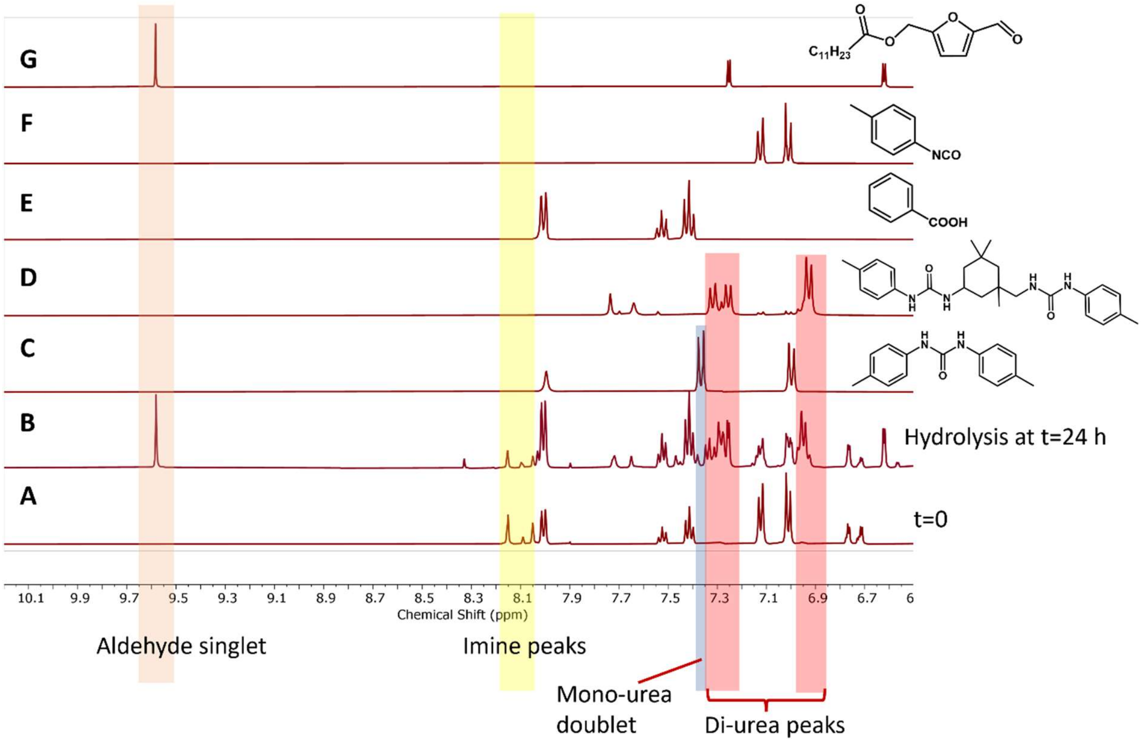

Figure S21. ^{1}H NMR spectra of hydrolysis product of **ALD3** in the presence of water and p-tolylisocyanate with benzoic acid as a catalyst at t=0 (A) and after 24 h (B), mono-urea compound synthesized through the reaction of p-tolylisocyanate and excess water (C), di-urea compound synthesized through the reaction of p-tolylisocyanate and isophorone diamine (D), benzoic acid (E), p-tolylisocyanate (F), and **A1** (G) recorded in THF-d_8. The ^{1}H NMR comparison shows that di-urea formation product (product D) outpaces the mono-urea formation product (product E) when employing benzoic acid catalysis.

5) Visual Appearance of the Cured Prepolymers

Table S2. Visual appearance of the prepolymers after curing at 60% relative humidity.

Prepolymers	Cured without benzoic acid catalysis	Cured with benzoic acid catalysis
**PPR1**	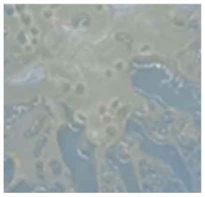	
**PP1**	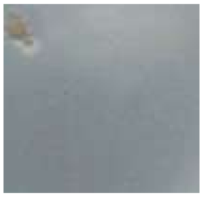	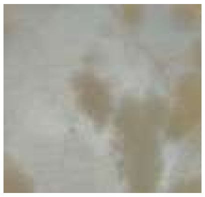
**PP2**	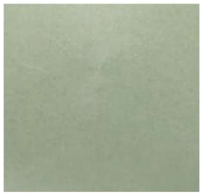	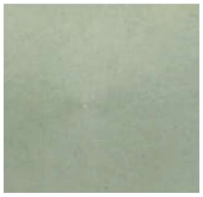
**PP3**	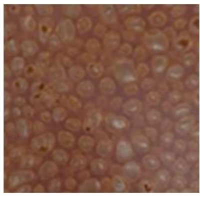	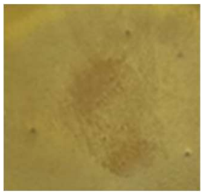
**PP4**	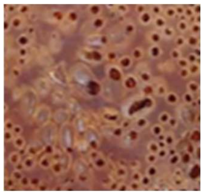	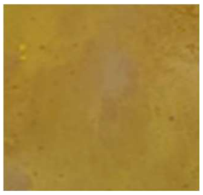
**PPR2**	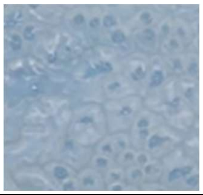	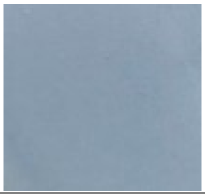

6) Tensile Stress-Strain Experiments

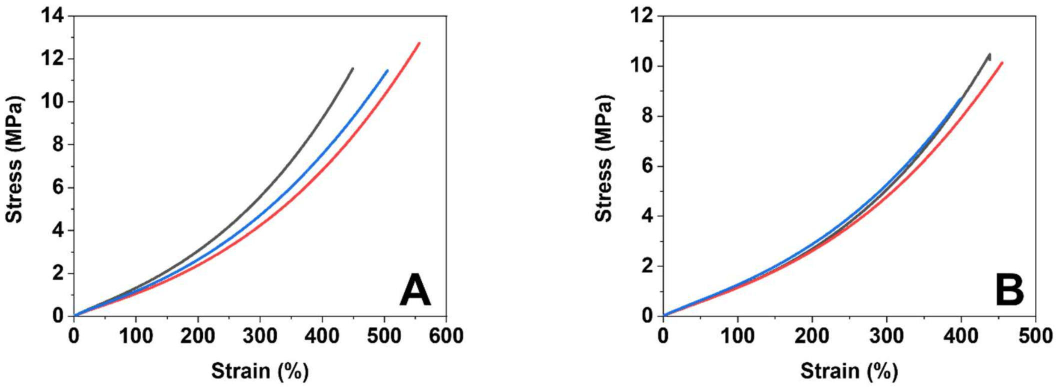

Figure S22. Tensile stress-strain curves for **PP1** after 1 week of curing under 60% relative humidity in the absence (A) in the presence (B) of benzoic acid catalysis.

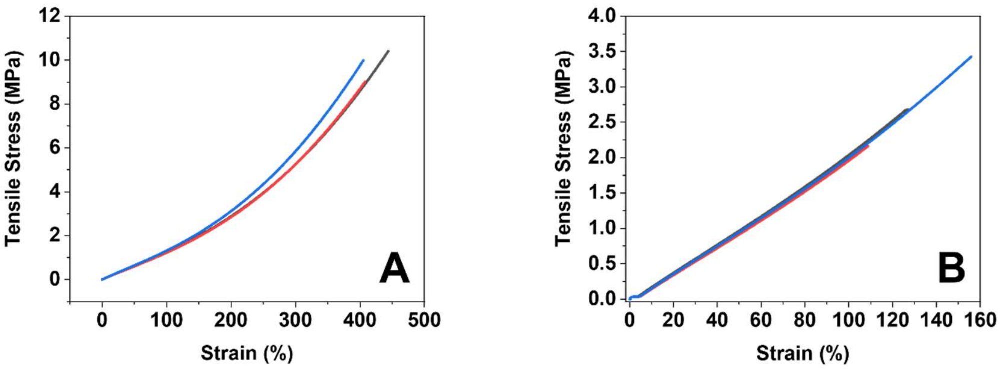

Figure S23. Tensile stress-strain curves for **PP2** after 1 week of curing under 60% relative humidity in the absence (A) in the presence (B) of benzoic acid catalysis.

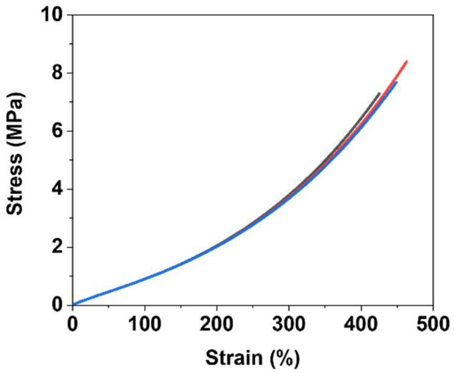

Figure S24. Tensile stress-strain curves for **PP3** after 1 week of curing under 60% relative humidity in the presence of benzoic acid catalysis.

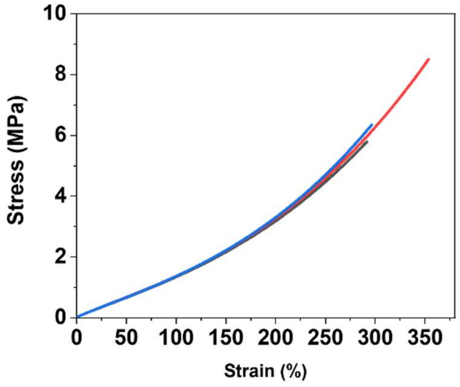

Figure S25. Tensile stress-strain curves for **PP4** after 1 week of curing under 60% relative humidity in the presence of benzoic acid catalysis.

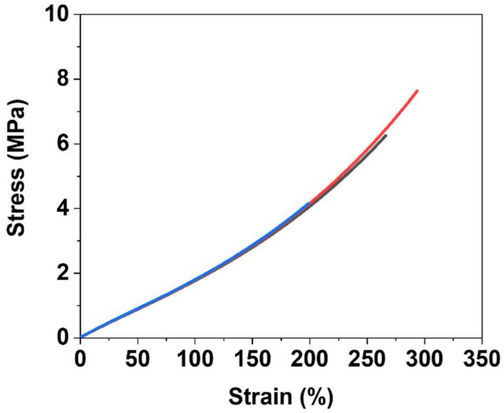

Figure S26. Tensile stress-strain curves for **PPR2** after 1 week of curing under 60% relative humidity in the presence of benzoic acid catalysis.

7) Dynamic Mechanical Analysis

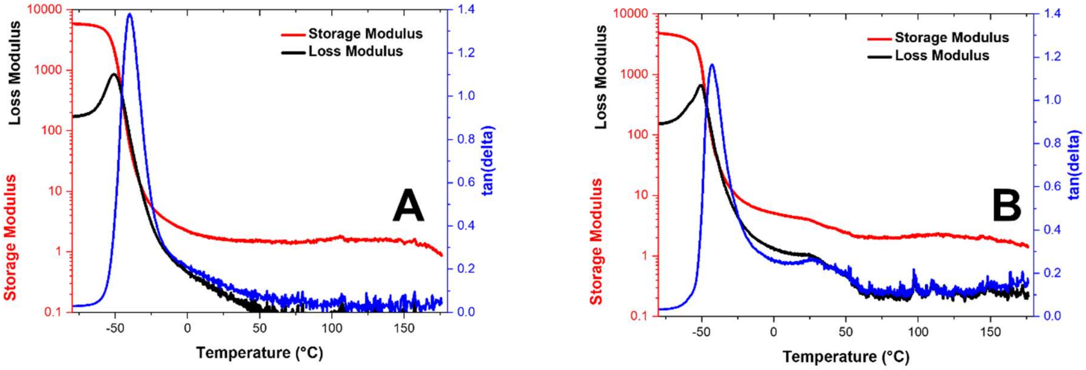

Figure S27. Dynamic mechanical analysis of **PP1** after 1 week of curing under 60% relative humidity in the absence (A) in the presence (B) of benzoic acid catalysis.

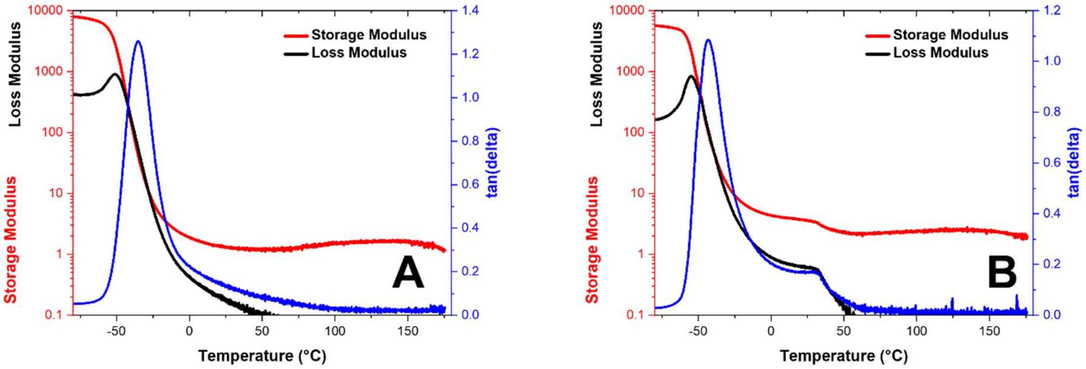

Figure S28. Dynamic mechanical analysis of **PP2** after 1 week of curing under 60% relative humidity in the absence (A) in the presence (B) of benzoic acid catalysis.

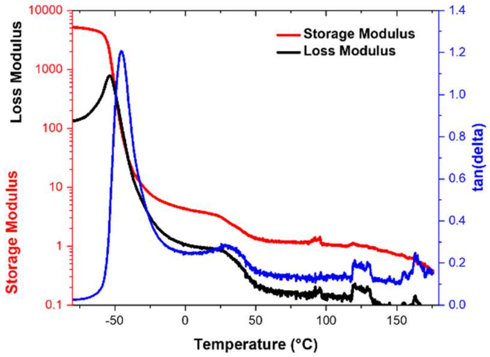

Figure S29. Dynamic mechanical analysis of **PP3** after 1 week of curing under 60% relative humidity in the presence of benzoic acid catalysis.

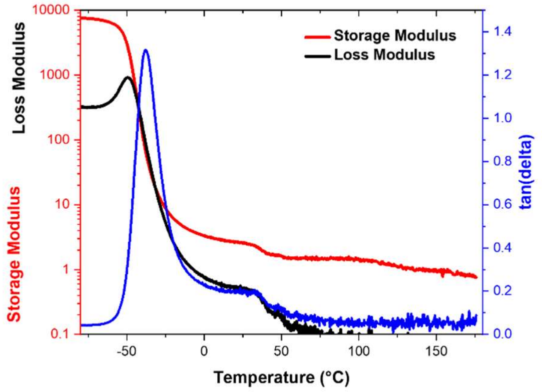

Figure S30. Dynamic mechanical analysis of **PP4** after 1 week of curing under 60% relative humidity in the presence of benzoic acid catalysis.

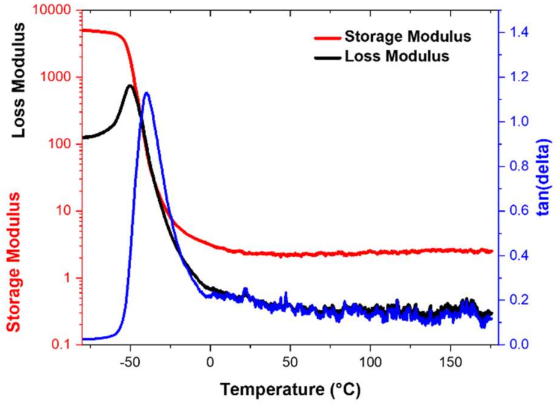

Figure S31. Dynamic mechanical analysis of **PPR2** after 1 week of curing under 60% relative humidity in the presence of benzoic acid catalysis.

8) Analysis of the Extracts & Networks After Extraction

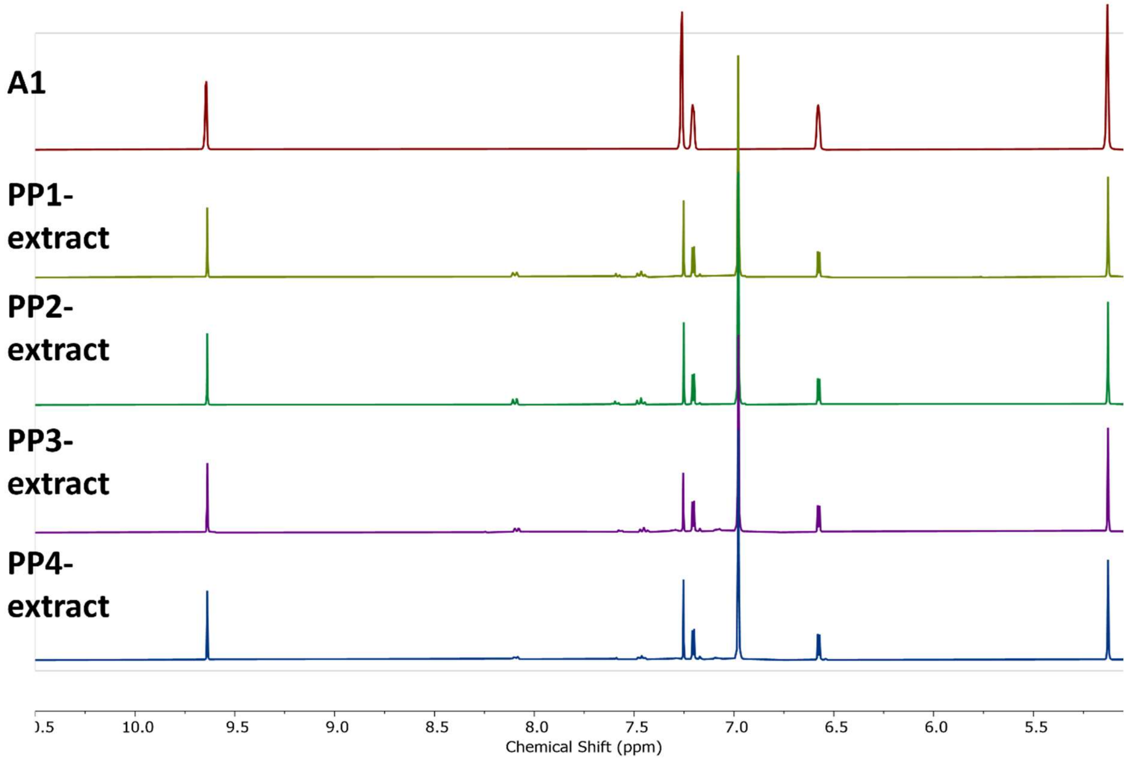

Figure S32. ^{1}H NMR analysis of the THF-extracts of the cured prepolymers vs. aldehyde **A1**, recorded in $CDCl_3$.

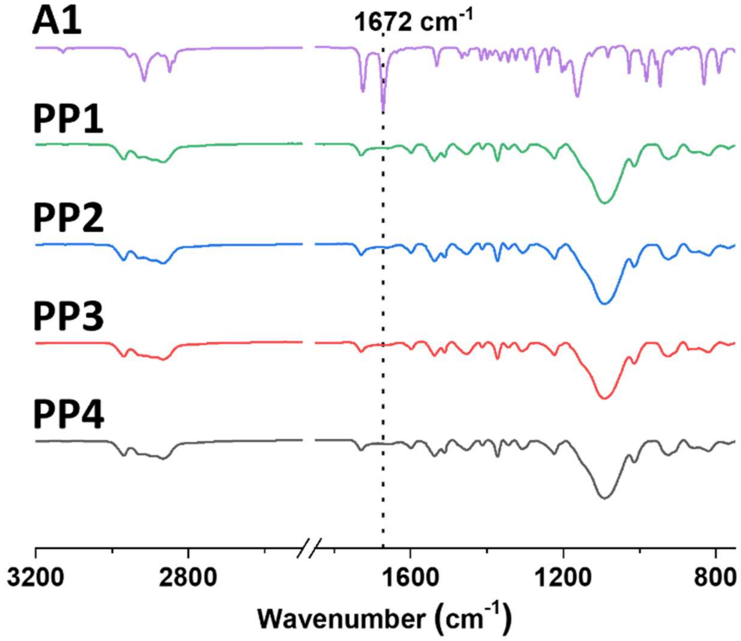

Figure S33. FT-IR analysis of **PP1**, **PP2**, **PP3** and **PP4** after extraction from THF.

9) Dynamic Mechanical Analysis of Extracted Networks

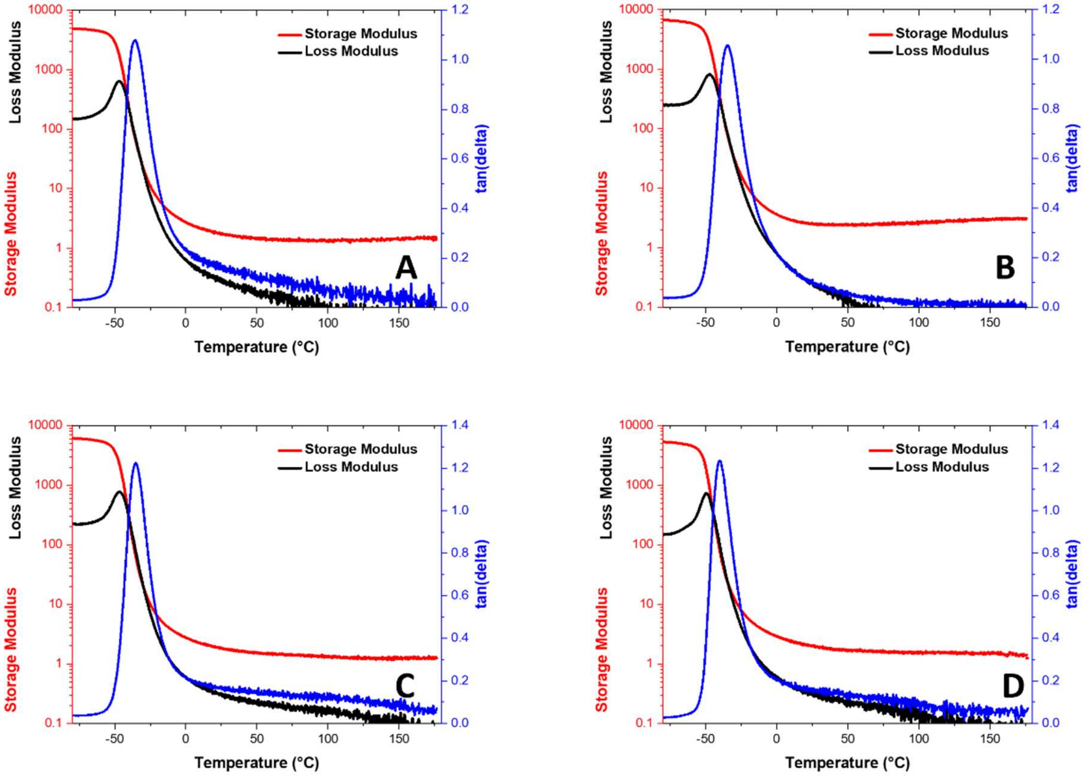

Figure S34. Dynamic mechanical analysis of **PP1** (A), **PP2** (B), **PP3** (C) and **PP4** (D) after extraction from THF.

10) Lap-Shear Tests

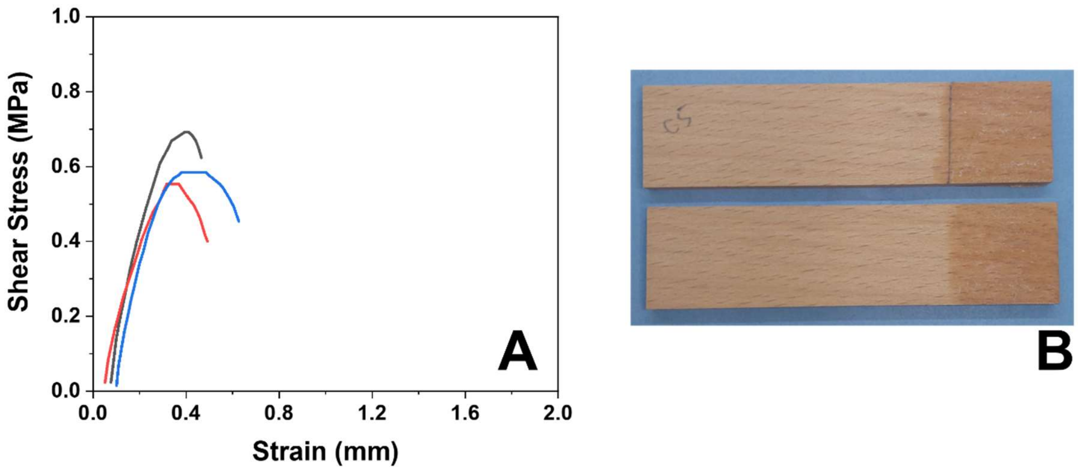

Figure S35. Shear stress-strain curves of prepolymer **PPR1** based adhesive after one week of curing under 60% relative humidity (A). Visual examination of shear surfaces of the adhesive applied (B).

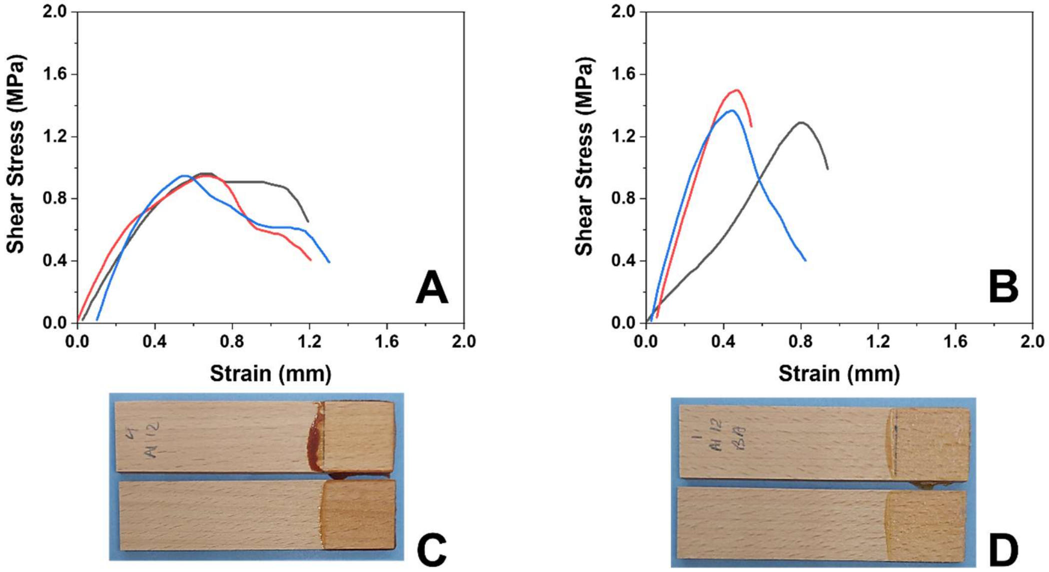

Figure S36. Shear stress-strain curves of **PP1** based adhesive after one week of curing under 60% relative humidity in the absence (A) and in the presence (B) of benzoic acid catalysis. Visual examination of shear surfaces of the adhesive applied in the absence (C) and in the presence (D) of benzoic acid catalysis.

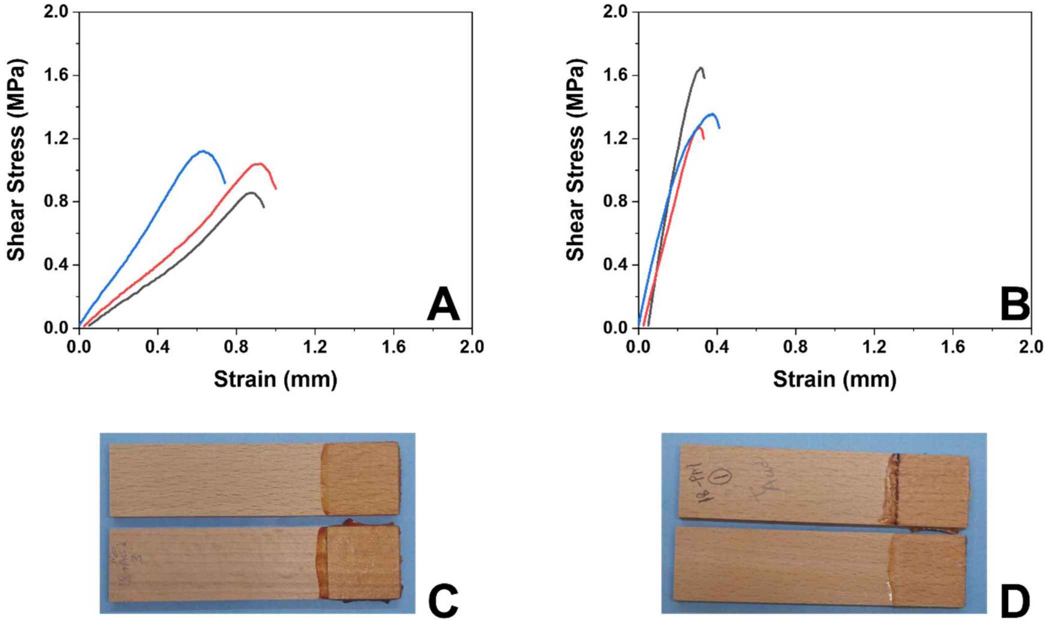

Figure S37. Shear stress-strain curves of **PP2** based adhesive after one week of curing under 60% relative humidity in the absence (A) and in the presence (B) of benzoic acid catalysis. Visual examination of shear surfaces of the adhesive applied in the absence (C) and in the presence (D) of benzoic acid catalysis.

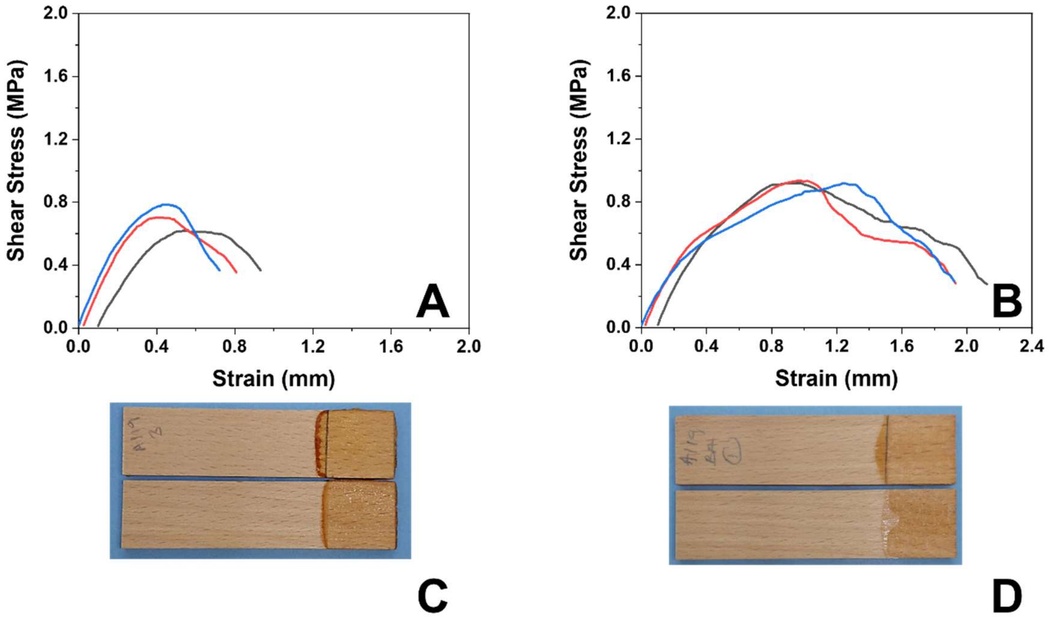

Figure S38. Shear stress-strain curves of **PP3** based adhesive after one week of curing under 60% relative humidity in the absence (A) and in the presence (B) of benzoic acid catalysis. Visual examination of shear surfaces of the adhesive applied in the absence (C) and in the presence (D) of benzoic acid catalysis.

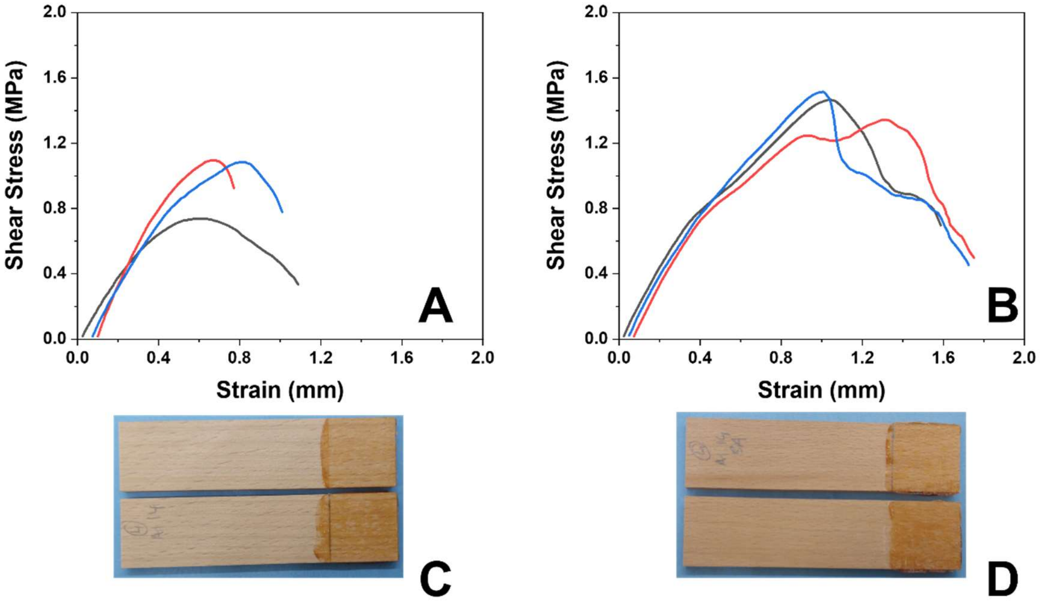

Figure S39. Shear stress-strain curves of **PP4** based adhesive after one week of curing under 60% relative humidity in the absence (A) and in the presence (B) of benzoic acid catalysis. Visual examination of shear surfaces of the adhesive applied in the absence (C) and in the presence (D) of benzoic acid catalysis.

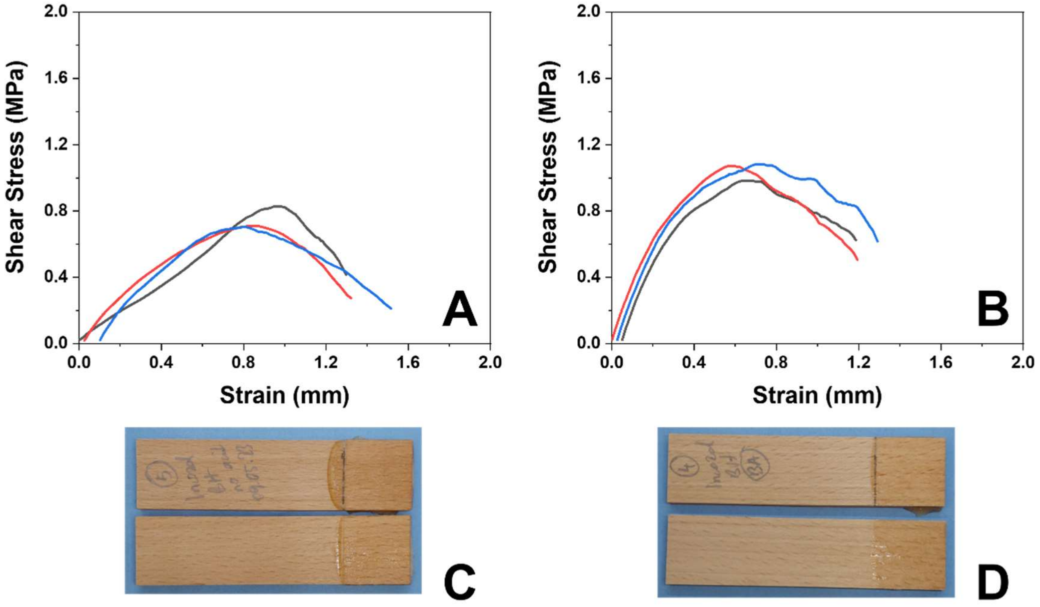

Figure S40. Shear stress-strain curves of **PPR2** based adhesive after one week of curing under 60% relative humidity in the absence (A) and in the presence (B) of benzoic acid catalysis. Visual examination of shear surfaces of the adhesive applied in the absence (C) and in the presence (D) of benzoic acid catalysis.
